# Longitudinal plasma proteomic analysis of 1117 hospitalized patients with COVID-19 identifies features associated with severity and outcomes

**DOI:** 10.1126/sciadv.adl5762

**Published:** 2024-05-24

**Authors:** Arthur Viode, Kinga K. Smolen, Patrick van Zalm, David Stevenson, Meenakshi Jha, Kenneth Parker, IMPACC Network, Ofer Levy, Judith A. Steen, Hanno Steen

**Affiliations:** ^1^ Department of Pathology, Boston Children’s Hospital, Boston, MA, USA.; ^2^ Harvard Medical School, Boston, MA, USA.; ^3^ Precision Vaccines Program, Boston Children’s Hospital, Boston, MA, USA.; ^4^ Department of Neuropsychology and Psychopharmacology, EURON, Faculty of Psychology and Neuroscience, Maastricht University, Maastricht, Netherlands.; ^5^ Broad Institute of MIT & Harvard, Cambridge, MA, USA.; ^6^ F. M. Kirby Neurobiology Center, Boston Children's Hospital, Boston, MA, USA.; ^7^ Neurobiology Program, Boston Children's Hospital, Boston, MA, USA.

## Abstract

Severe acute respiratory syndrome coronavirus 2 (SARS-CoV-2) infection is characterized by highly heterogeneous manifestations ranging from asymptomatic cases to death for still incompletely understood reasons. As part of the IMmunoPhenotyping Assessment in a COVID-19 Cohort study, we mapped the plasma proteomes of 1117 hospitalized patients with COVID-19 from 15 hospitals across the United States. Up to six samples were collected within ~28 days of hospitalization resulting in one of the largest COVID-19 plasma proteomics cohorts with 2934 samples. Using perchloric acid to deplete the most abundant plasma proteins allowed for detecting 2910 proteins. Our findings show that increased levels of neutrophil extracellular trap and heart damage markers are associated with fatal outcomes. Our analysis also identified prognostic biomarkers for worsening severity and death. Our comprehensive longitudinal plasma proteomics study, involving 1117 participants and 2934 samples, allowed for testing the generalizability of the findings of many previous COVID-19 plasma proteomics studies using much smaller cohorts.

## Teaser

Detecting 2910 proteins in 2934 COVID-19 plasmas identifies generalizable findings across many sites and many previous studies.

## INTRODUCTION

The COVID-19 pandemic has infected >700 million individuals and caused >7 million reported deaths worldwide (https://covid19.who.int/). COVID-19 is a disease with very heterogeneous symptomatology with most infected individuals displaying mild symptoms including cough, fever, and a sore throat (*1*). However, the virus is also responsible for severe disease manifestation characterized by acute respiratory distress syndrome, thrombosis, and multiorgan dysfunction, devastating human life and challenging health care systems worldwide.

While our comprehension of immune responses to severe acute respiratory syndrome coronavirus 2 (SARS-CoV-2) infection has grown tremendously since it was first described in December 2019, the challenge of prognosticating clinical disease trajectory, disease severity, and outcome of hospitalized patients with COVID-19 remains. Understanding changes in the plasma proteome associated with specific disease trajectories and/or outcomes may inform our evolving management options and approaches to SARS-CoV-2 as well as future emerging viral infections.

Several studies investigating the immuno-proteomic response to COVID-19 have presented notable differences between hospitalized cases and controls (*2*–*23*). Severe disease outcomes are characterized by immune dysregulation and an excessive inflammatory state due to endothelial cell activation or dysfunction (*24*–*26*). Notably, interleukin-6 (IL-6) and the IL-6–inducible C-reactive protein, two major immune mediators of inflammation, are increased in the plasma of patients with COVID-19 (*27*). Although proinflammatory cytokines are elevated, SARS-CoV-2 suppresses type 1 interferon responses key for induction of antiviral defenses of neighboring cells and the adaptive immune system (*28*, *29*). The dysregulation of the complement system then leads to adverse effects (*30*), including excessive activation of neutrophils resulting in the release of neutrophil extracellular traps (NETS) (*31*–*35*) displaying antimicrobial and cytotoxic extracellular histones. These events lead to inflammation, immune thrombosis (*32*), and organ damage (*36*–*38*). The pathogenesis of SARS-CoV-2 infection is complex, and while certain aspects are well described, the kinetics of mechanisms leading to disease severity and heterogeneity are incompletely understood. In particular, the temporal changes to the plasma proteome during the hospital stay are understudied because of the scarcity of studies that have gathered longitudinal samples (*6*, *7*, *9*, *16*, *17*). Furthermore, most of the collected samples originate from a single-center cohort, which might limit the generalizability of the findings in the plethora of earlier studies on much smaller cohorts. A comprehensive review of the current state of COVID-19 proteomics can be found in table S1.

Within the National Institutes of Health (NIH)/National Institute of Allergy and Infectious Diseases (NIAID)–funded IMmunoPhenotyping Assessment in a COVID-19 Cohort (IMPACC) (*39*, *40*), a multicenter longitudinal study focused on immunophenotyping hospitalized patients with COVID-19 across the United States, we mapped the proteomes of 2934 longitudinal plasma samples obtained from 1117 study participants, each of whom provided up to 6 plasma samples during their hospitalization. Detailed information about chronological distribution of the samples and the number of samples per time point can be found in fig. S1 and table S2. Hence, each patient served as his/her own control. These samples were collected at 15 clinical sites across the United States between May 2020 and April 2021, before widely available vaccination and/or widespread circulation of emerging variants.

Because of restrictions in the early phase of the COVID-19 pandemic, samples from healthy donors were not collected as the clinical sites did not permit the inclusion of healthy individuals in research studies. Enrollment of healthy individuals as matched controls [based on age, sex, and or body mass index (BMI)] was then considered an unacceptable health risk. In addition, healthy controls would have been only of limited use for the IMPACC study, which was designed to map the immunophenotypic heterogeneity within a hospitalized COVID-19 cohort to better understand the underpinnings of the different manifestations of COVID-19 observed in this patient population. The patients were stratified into five clinical trajectory groups (TGs) based on disease severity, ranging from those with short (i.e., <3 days) hospital stay (TG1) to those with fatal outcomes within 28 days (TG5) (*39*, *40*).

We used a biochemical method to deplete the most abundant plasma proteins followed by liquid chromatography–mass spectrometry (LC-MS)–based discovery proteomics to characterize TG-associated longitudinal plasma proteome signatures. To overcome the challenges linked to the deep analysis of thousands of plasma samples, we developed a highly reproducible high-throughput plasma proteomic platform encompassing sample preparation, sample analysis, and data analysis (*41*). By depleting highly abundant plasma proteins using perchloric acid (*42*–*44*), we achieved exceptional levels of analytical depth, robustness, cost efficiency, and throughput.

In summary, we examine the temporal changes in the plasma proteome associated with COVID-19 disease severity by applying our high-throughput LC-MS–based proteomics platform within the well-powered IMPACC cohort. This study represents one of the largest LC-MS–based proteomics studies on depleted plasma to date. By analyzing 2934 plasma samples from 1117 patients with COVID-19 during their hospital stay, we provide robust insights into the changes in the plasma proteome associated with disease severity and their temporal changes across 15 clinical centers. Our findings specifically highlight an association of NETs and heart damage markers in the most severe cases of COVID-19 with fatal outcome. Furthermore, we provide a comprehensive description of the temporal ordering of plasma proteome changes associated with fatal outcomes. We can identify both general and virus-specific plasma proteome signatures by comparing our findings with a recent plasma proteomics study on Ebola infection. Our longitudinal, multicenter cohort data are a valuable and widely applicable resource for informing decisions about the initiation of therapeutic interventions.

## RESULTS

### High-throughput deep plasma proteomic mapping

**Fig. 1.**
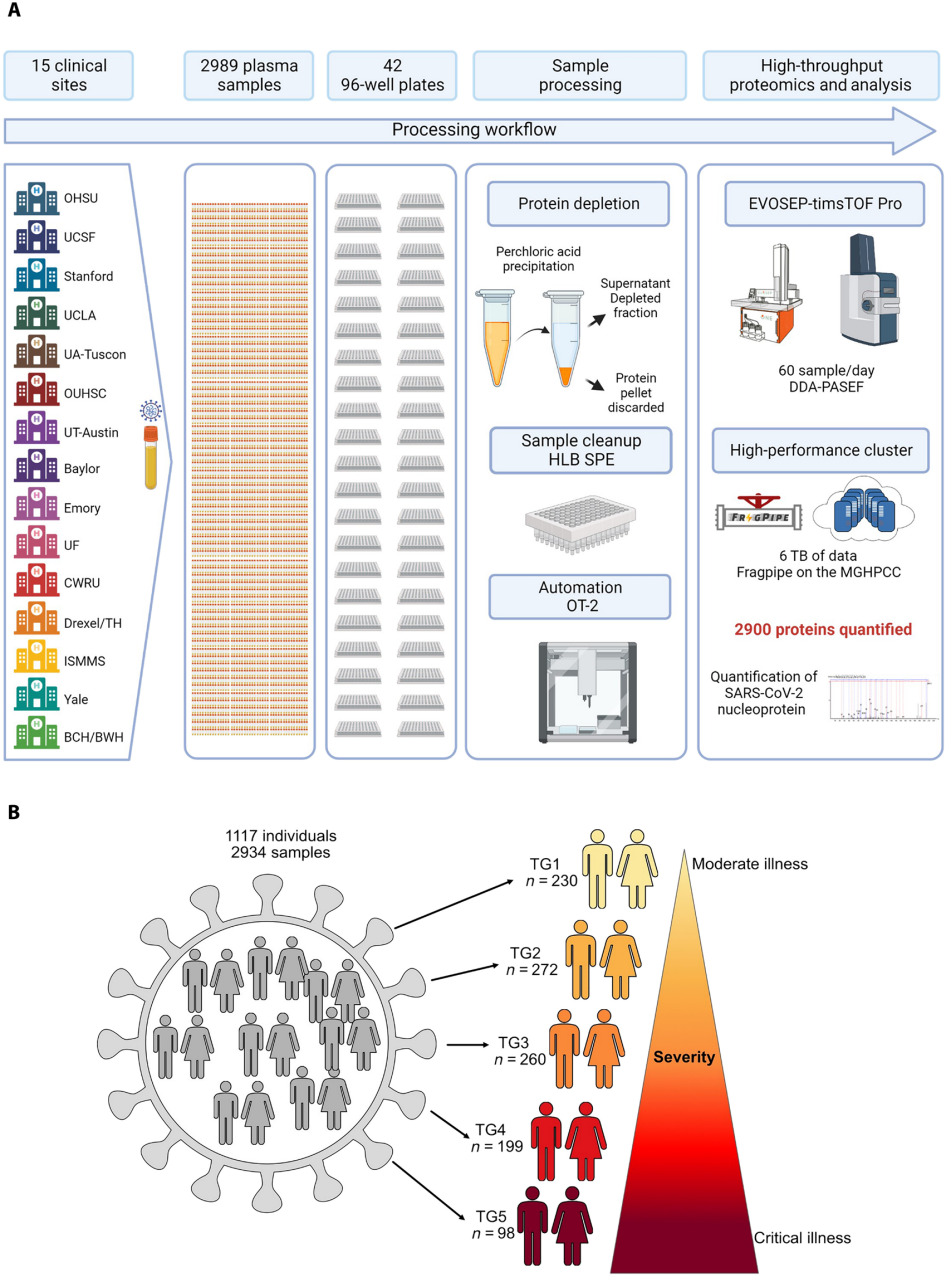
High-throughput LCMS–based method for the analysis of thousands of plasma samples and cohort design. (**A**) Workflow: Perchloric acid plasma proteomic platform. (**B**) Individuals were categorized into five clinical TGs based on disease severity. TG1, TG2, TG3, TG4, and TG5 from moderate illness to critical illness. TG5 comprises individuals who died within 28 days of hospitalization.

As part of the IMPACC study (*39*), we analyzed 2934 plasma samples longitudinally collected from 1117 hospitalized individuals, with polymerase chain reaction (PCR)–confirmed SARS-CoV-2 infection (Fig. 1A), using our in-house developed high-throughput deep plasma proteomic pipeline. The breakdown of the plasma samples can be found in table S2 (*39*). The multicenter design provided an unprecedented number of samples, which allowed for a generalizable in-depth analysis of the plasma proteomes associated with COVID-19 severity and heterogeneity of hospitalized patients, making this study one of the largest COVID-19 cohorts analyzed by MS-based discovery proteomics to date. The study’s primary aim was to conduct a comprehensive immunophenotyping analysis of hospitalized patients with COVID-19 with varying degrees of disease severity. By elucidating the immunological mechanisms associated with different severities of COVID-19, this research sought to enhance our understanding of the disease and facilitate the development of more effective prognostic strategies and therapeutic interventions based on actionable biomarkers. Furthermore, the samples were collected before any widely available vaccinations and/or the emergence of variants (*40*), providing a unique opportunity to investigate the immune response of patients immunologically naïve toward SARS-CoV-2 or SARS-CoV-2–derived antigens.

Patients were classified by the clinicians of this IMPACC study into five clinical TGs reflecting both the degree of respiratory support required during the hospitalization and the presence or absence of activity limitations or oxygen requirement at discharge (Fig. 1B); details and rationale for this stratification can be found in (*40*). Longitudinal patient distribution can be found in table S2. The different severity-based TGs showed similar distributions across the 15 clinical institutions as illustrated in table S3. In addition to the fatalities of TG5, 32 patients in TG4 (*n* = 32 of 199, 16.1%) died after 28 days of hospitalization, i.e., after the end of the hospitalization phase of the study period. Detailed patient characteristics (sex, age, respiratory status upon admission, outcome after 28 days, BMI, and symptom onset) can be found in table S4. Table S4 contains demographic information, including breakdown by sex (39.9% female: 60.1% male), age (median: 59 years of age), BMI (median: 31.3), and symptom onset (median: 7 days). We performed a principal components analysis (PCA) to determine whether a site-dependent (fig. S3A) batch effect is observable; the superposition of the 95%-confidence ellipses indicates good robustness of the sample collection and processing across the different enrollment sites. In comparison, we also performed a PCA (fig. S3B) based on the TG, which shows a clear TG-dependent shift in the position of the 95%-confidence ellipses.

**Fig. 2.**
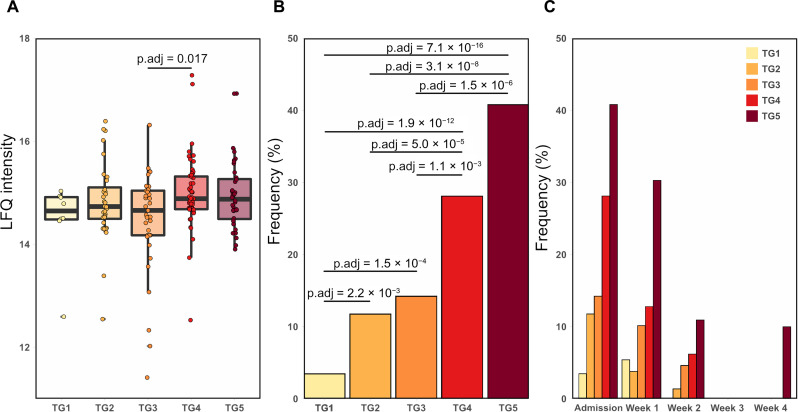
Frequency of detection of SARS-CoV-2 NP in plasma was associated with COVID-19 severity. (**A**) Boxplot of the Label-free quantification (LFQ) intensity of the N protein at admission. We performed a one-way analysis of variance (ANOVA) and found statistical differences with an adjusted *P* value of 0.034. A post hoc test was done, a statistical difference was found between TG3 versus TG4 (*P* value = 0.0172). (**B**) Frequency of SARS-CoV-2–derived NP at admission to the hospital for each TG. The frequency of NP detection for each TG was as follows: TG1 had 3% (8 of 230), TG2 12% (32 of 272), TG3 14% (37 of 260), TG4 28% (56 of 199), and TG5 41% (40 of 98). We applied a Fisher’s exact test to assess statistical differences between TGs. Frequency NCAP: statistically highly significant TG1/TG2 (*P*_adj_ = 2.2 × 10^−3^), TG1/TG3 (*P*_adj_ = 1.5 × 10^−4^), TG1/TG4 (*P*_adj_ = 1.9 × 10^−12^), TG1/TG5 (*P*_adj_ = 7.1 × 10^−16^), TG2/TG4 (*P*_adj_ = 5.0 × 10^−5^), TG2/TG5 (*P*_adj_ = 3.1 × 10^−8^), TG3/TG4 (*P*_adj_ = 1.1 × 10^−3^), TG3/TG5 (*P*_adj_ = 1.5 × 10^−6^). (**C**) Frequency of SARS-CoV-2–derived NP at admission to the hospital, week 1, week 2, week 3, and week 4 for each TG. At week 4, the NP was only observed in one individual belonging to TG5. No NP was observed after 4 weeks of hospitalization.

We used an unbiased LC-MS–based high-throughput plasma proteome method to analyze this large cohort and query the tissue leakage proteome found in blood plasma. A challenge of plasma proteomics is that the 22 most abundant plasma proteins account for ~99% of the total plasma protein content (*45*) affecting the ability to detect proteins of lower abundance. To overcome this challenge, we advanced our perchloric acid–based precipitation protocol (*42*–*44*) to remove cost efficiently, reproducibly, and efficiently the high-abundance plasma proteins and detect low-abundant proteins such as viral protein (Fig. 2A). This approach detected and quantified 2910 proteins in plasma including SARS-CoV-2–derived proteins (*44*) even at a throughput of 60 samples per day. On average, 576 proteins were quantified per sample, and 532 proteins have less than 50% missing values, i.e., were identified in more than 1467 samples (fig. S3C).

### Identification of SARS-CoV-2–derived nucleoprotein in plasma

The depth of our analysis is exemplified by the detection of the nucleoprotein (NP) from SARS-CoV-2. NP is the most abundant SARS-CoV-2–derived protein in other COVID-19 proteomics studies (*46*–*48*) focusing on either SARS-CoV-2 samples or samples from body fluids known to be enriched in viral particles. The SARS-CoV-2–derived NP was identified in 287 individual samples based on a total of nine peptides corresponding to a cumulative sequence coverage of 29% (fig. S4A). Example tandem mass spectra of the precursor ion NPANNAAIVLQLPQGTTLPK (z = 2+) can be found in fig. S4B. We observed small, but significant changes in the abundance of the NP between TG3 and both TG4 and TG5 at admission (*P*_adj_ of 0.011 and 0.028, respectively) (Fig. 2A). However, more prominent changes become apparent when performing a Fisher’s exact test to compare the observation frequency of NP between the five TGs at admission: A significantly higher frequency of NP was detected in severe COVID-19 cases, i.e., TG4 and TG5 in comparison to the less severe TGs: in TG4 and TG5, NP was detected in 30 to 40% of the TG4 and TG5 samples, in contrast to only 3 to 14% of TG1 to TG3 samples (Fig. 2, B and C). The frequency of observation of the NP markedly diminished during the first 2 weeks of hospitalization and was detectable in only a single sample past 2 weeks of hospitalization.

### Severity group–dependent immune response signatures at time of admission

**Fig. 3.**
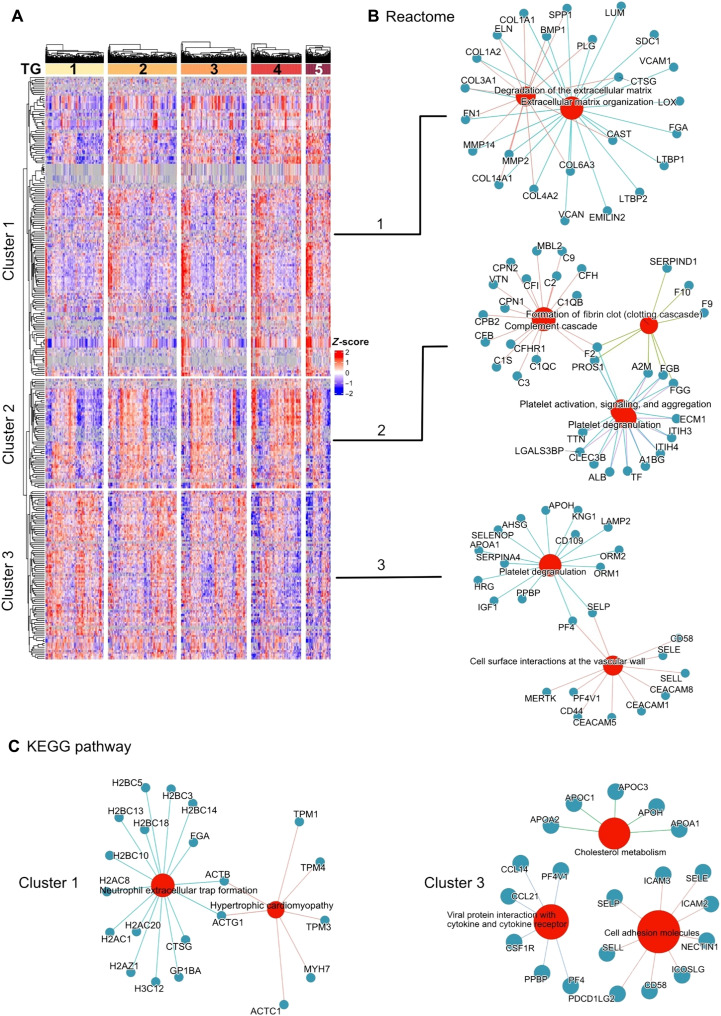
Immune response signatures at hospital admission differentiated between severity groups. (**A**) Hierarchical clustering of the 305 proteins exhibiting differential abundances among the five TGs, based on one-way ANOVA. Each row represents a protein, and each cell represents the protein abundance (*Z*-scored) (the red represents an up-regulation; blue represents a down-regulation; gray represents missing values). Samples were ordered on the basis of increased severity from the mildest (TG1) to the most severe group (TG5). We observed three clusters. (**B**) Reactome pathway enrichment analysis. Cluster 1: enrichment for ECM organization and degradation of the ECM. Cluster 2: enriched for innate immune system, complement cascade, formation of fibrin clot (clotting cascade), and platelet degranulation. Cluster 3: enrichment for platelet degranulation and cell surface interaction at the vascular wall. (**C**) KEGG pathway enrichment analysis. Cluster 1: enrichment for NET formation and cardiomyopathies. Cluster 3: enrichment for cell adhesion molecules, viral protein interaction with cytokine and cytokine receptor, and cholesterol metabolism.

In line with the overall IMPACC objectives, we focused on characterizing the severity-dependent differences in the host immunophenotype of hospitalized patients with COVID-19. To this end, we characterized the early host response to SARS-CoV-2 infection upon hospital admission. We performed a one-way analysis of variance (ANOVA) to identify TG-dependent proteins abundance changes. After Benjamini-Hochberg (BH) multiple-testing correction, we identified 305 proteins of significant difference in abundance (*P*_adj_ < 0.05). These proteins were further analyzed using an unsupervised hierarchical clustering approach. The associated heatmap (Fig. 3A) shows three main protein clusters with profound differences between the TGs upon admission to the hospital (post hoc Tukey test; table S5). Cluster 1 reveals 158 proteins whose expression levels rise with severity with a more pronounced increase toward the severe TGs. Cluster 2 describes 58 proteins whose expression levels are particularly low in TG1 in comparison to TG2 to TG5. Cluster 3 describes 89 proteins whose expression levels decreased with severity. Each cluster was submitted to separate pathway enrichment analyses.

The bioinformatic analysis for Cluster 1 (Fig. 3, B and C) revealed several biological Kyoto Encyclopedia of Genes and Genomes (KEGG) and Reactome pathways correlated with disease severity (TG4 and TG5), i.e., up-regulation of proteins levels with more severe TGs. These proteins are linked to NET formation (*P*_adj_ = 6.7 × 10^−7^), extracellular matrix (ECM) organization (*P*_adj_ = 5.3 × 10^−6^), degradation of the ECM (*P*_adj_ = 4.3 × 10^−5^), and hypertrophic and/or dilated cardiomyopathies (*P*_adj_ = 4.2 × 10^−2^). The pathways hypertrophic and dilated cardiomyopathy will be referred to as cardiomyopathy throughout the text for simplicity. Proteins linked to NET formation are for example core histones H2 (H2AC8) and cathepsin G (CTSG). Proteins linked to cardiomyopathy include Myosin-7 (MYH7) and Tropomyosin 3 (TPM3). The pathway degradation of the ECM is composed of proteins such as matrix metallopeptidase 14 (MMP14) and Elastin (ELN).

Cluster 2 (Fig. 3B) revealed protein associated with an acute infection state (lower in TG1, high in all the other TGs). Biological pathways involved in innate immune system (*P*_adj_ = 3.4 × 10^−5^), complement cascade (*P*_adj_ = 2.7 × 10^−15^), formation of fibrin clot (*P*_adj_ = 1.7 × 10^−5^), and platelet degranulation (*P*_adj_ = 1.2 × 10^−5^) were identified. In these pathways, we identified several complement proteins such as Complement 2 (C2), Complement C1q subcomponent subunit C (C1QC), and fibrinogens B and G (FGB and FGG).

Cluster 3 (Fig. 3, B and C) revealed proteins whose levels increased with milder COVID-19, i.e., proteins linked to cholesterol metabolism [*P*_adj_ = 1.8 × 10^−2^; example: apolipoprotein A1 (APOA1)], cell adhesion molecules [*P*_adj_ = 1.3 × 10^−3^; example E-selectin (SELE)], viral protein interaction with cytokine and cytokine receptor [*P*_adj_ = 1.5 × 10^−3^; example: platelet factor 4 (PF4)], and platelet degranulation (*P*_adj_ = 1.7 × 10^−13^; example: platelet basic protein (PPBP)].

### COVID-19 progression/outcome-associated signatures in plasma

**Fig. 4.**
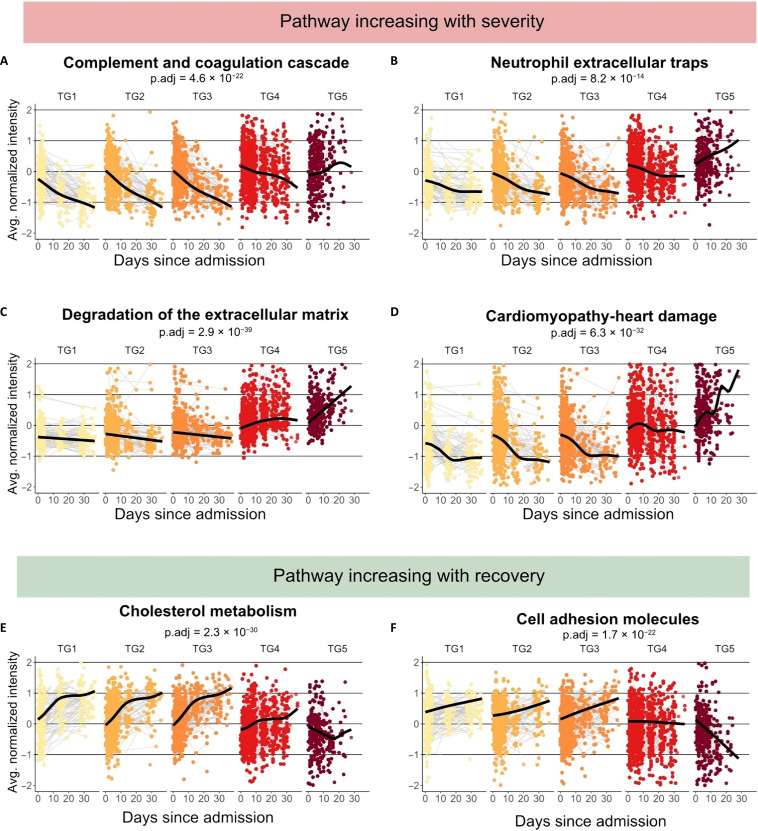
Longitudinal study of the pathways dysregulated at admission. Time course analysis of selected biological pathways. A generalized additive model with mixed effects analysis to evaluate the effects of the TG was performed. Enrollment sites and individuals were considered random effects. TGs, age, and sex were considered as fixed effects. The TGs are plotted from mild (light yellow) to severe (dark red). We highlighted pathways increasing over time in TG5 and decreasing in the other TGs such as (**A**) complement and coagulation cascades, (**B**) NETs, (**C**) degradation of the ECM, and those associated with (**D**) cardiomyopathies. Pathways decreasing over time in TG5 and increasing in the other TGs were plotted: (**E**) cholesterol metabolism and (**F**) cell adhesion molecules. Corresponding *P* values can be found in table S2.

Subsequently, we explored the trajectories of biological pathways involved in COVID-19 pathophysiology throughout the hospitalization period, starting at admission (Fig. 4, additional detailed information can be found in table S4). On the basis of the baseline data at admission, we used smoothing spline regression analysis to interrogate the temporal trajectories of previously identified biological pathways. The biological pathways associated with poor outcome, i.e., those increasing with time and severity (TG5), are linked to inflammation and innate immune response, including complement and coagulation cascades (Fig. 4A), NETs (Fig. 4B), degradation of the ECM (Fig. 4C), and organ damage (Fig. 4D). The *P* values of the shape (smoothing term) and average (intercept) are listed in table S6.

At admission, the complement and coagulation cascade pathways have similar expression levels in all clinical TGs (Fig. 4A). Expression levels of the complement and coagulation cascades diverge on day 10 between mild and severe COVID-19 cases. Expression levels in TG1 to TG3 continuously decrease over time, with TG2 and TG3 expression levels converging with TG1 within 1 month after admission, i.e., at the end of the hospitalization phase of the study period. While TG4 also decreases over time, its expression level remained higher than those of TG1 to TG3. In contrast, TG5 expression levels start out even lower than TG3 but quickly increase with time surpassing TG4 at ~10 days after admission.

The expression levels of proteins linked to NETs (Fig. 4B) follow similar trajectories as the one described for the complement and coagulation cascades, with the major difference that TG5 demonstrated an elevated level similar to TG4 as early as admission to the hospital. Expression levels of TG5 sharply increase with time within the first week of admission to the hospital and rapidly differentiate from TG4, which shows a continuous decline, though TG4 stays consistently up-regulated in comparison with TG1 to TG3. Expression levels of TG2 and TG3 converge reaching similar levels as TG1 at ~20 days.

Similar to the NET-associated proteins, the degradation of ECM (Fig. 4C) was characterized by a sharp increase of the expression levels in TG5 and a decrease of the expression levels in TG1 to TG3. The TG4 expression levels initially increase with time as did TG5, albeit at a lower rate, to level off and decline after day ~22.

A more pronounced increase compared to the aforementioned pathways, is observed for proteins associated with the “organ damage” KEGG pathway (Fig. 4D). Consistent with the analysis of the plasma proteomes at admission, cardiomyopathy proteins were elevated in TG4 and TG5. In addition, they displayed the sharpest expression increase in TG5, while the expression levels of TG1 to TG3 decreased with time to reach a steady level at ~18 days. Proteins associated with this pathway are heart-specific proteins such as MYH7 or muscle proteins such as TMP1 and actin ACTB.

We also identified several biological pathways, whose increases over time were associated with positive outcomes and resolution of the disease (TG1, TG2, and TG3). The biological pathway of cholesterol metabolism (Fig. 4E), a marker of liver health, demonstrated increased expression levels with time in all TGs but TG5, indicating progressive severe liver/organ damage in this most severe TG. Increasing expression levels of TG2 and TG3 converge with TG1 at ~2 weeks after admission reflecting the recovery of the liver function. TG4 also demonstrated improving liver function over time, albeit at a lower level and slower rate of improvement. Similarly, the KEGG pathway cell adhesion molecules (Fig. 4F) drastically decreased over time in TG5, while increasing in TG1 to TG3. TG4 expression levels remained constant.

In summary, the biological pathways described above highlight the importance of longitudinal sampling as early trajectory information between the first two sampling time points are often more informative than the single sample taken, e.g., at admission. This pattern is repeatedly strengthened by the observation that TG5 shows repeatedly divergent temporal trajectories from other TGs. While TG2 and TG3 have similar expression levels and converge with TG1 after 2 to 3 weeks. The TG4 trajectories tend to fall between the trajectories of the less severe TG1 to TG3 and the fatal TG5.

### Immunophenotypic plasma protein signatures for death and collateral damage

**Fig. 5.**
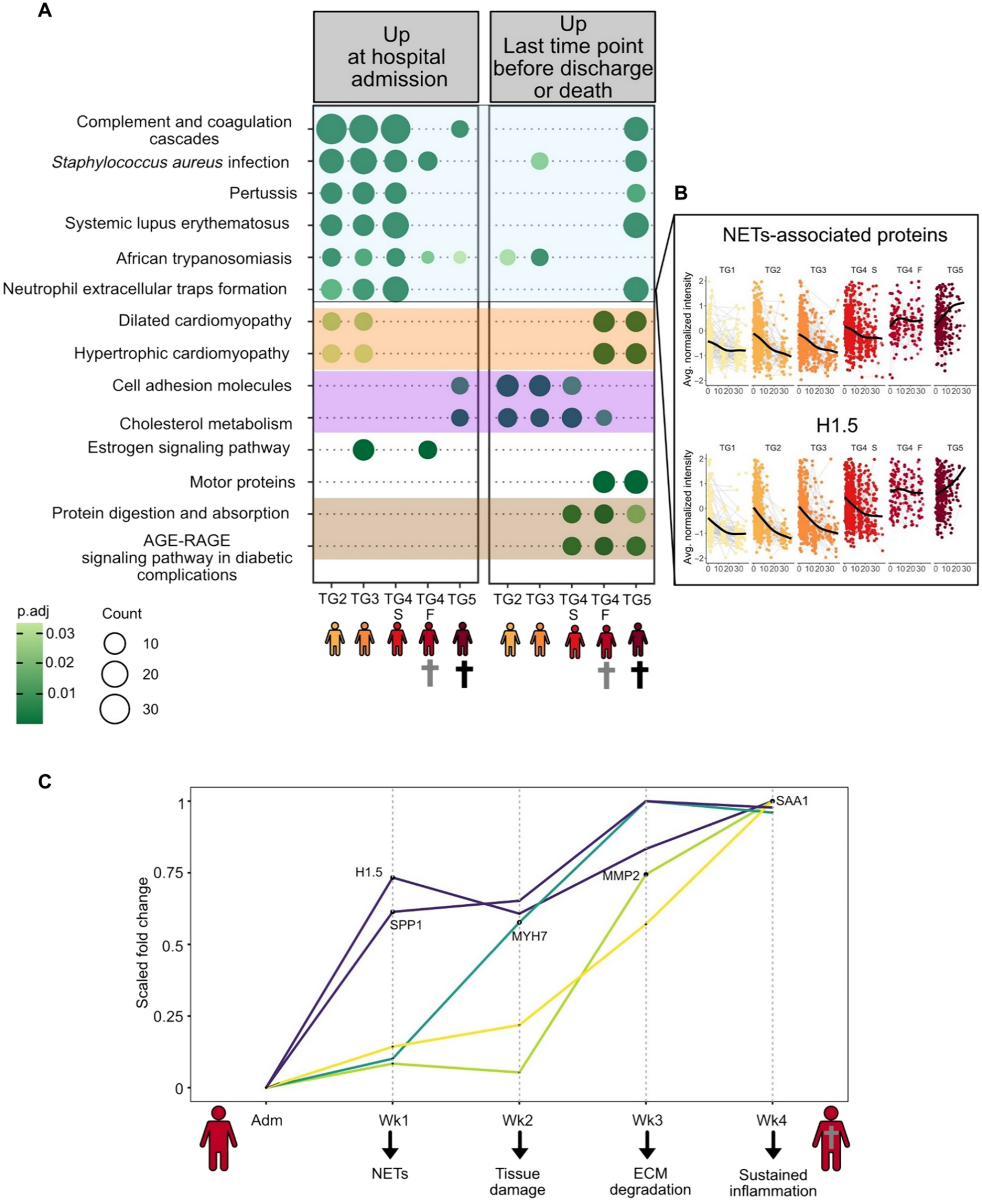
Death signature and collateral damage – sequence of events. (**A**) Our analysis focused on comparison between the first and last time points collected per individual. For better understanding of proteomic signatures associated with death, we split TG4 into two subgroups: TG4 survivors (TG4-S) and TG4 fatalities (TG4-F). TG4-F represents those who eventually died within the study but only after the 28-day period. TG4-F included 32 individuals (16%) who died within TG4. In contrast, TG5 is uniquely composed of individuals who died within 28 days. A paired *t* test (first sample collected versus last sample collected) was performed for each TG. Significant proteins were subjected to KEGG pathway enrichment analysis. Significant pathways were plotted using the compareCluster function of Cluster Profiler. (**B**) Highlights the NETs-associated proteins pathway and the protein H1 to H5 belonging to that pathway. (**C**) Longitudinal changes between TG4-S and TG4-F were mapped. To do so, *t* tests were performed at admission, week 1, week 2, week 3, and week 4. We identified key proteins whose abundances are changing over time between TG4-S and TG4-F. The fold change was normalized for each protein from 0 to 1 (*y* axis). There are no significant differences at admission between TG4-S and TG4-F. As soon as the first week 1, H1.5 and SPP1 are up-regulated in TG4-F, then, MYH7 at week 2, MMP2 at week 3, and lastly, SAA1 at week 4.

We observed profound differences in immune related proteins between the TGs. To better understand the pathophysiology that differentiates survival and death from COVID-19, we split TG4 into two subgroups: TG4 survivors (TG4-S) and TG4 fatalities (TG4-F). TG4-F includes those 32 (16%) participants who eventually died within the study period but only after the 28-day period, which defined the assignment to TG5. We performed a paired *t* test between the first sample (admission) and the last sample collected for each individual before discharge from the hospital or death. BH-corrected significant proteins were submitted to biological pathway analysis using the ClusterProfiler R/Bioconductor package (*49*) (Fig. 5 and fig. S5). We observed a biological pathway profile unique to TG5, identifying a predictive “death profile” in hospitalized patients with COVID-19 (Fig. 5A, highlighted in blue). The predictive death profile signature is based on visit 1 (at admission) samples only. These pathways include, for example, complement and coagulation cascades and NETs formation. Figure 5B depicted the trajectory of the NETs formation pathway and the protein Histone H1.5 (H1.5). Further examples of proteins linked to these pathways and their trajectory overtime can be found in fig. S6.

Next, we examined biological pathways showing increased abundance levels in the fatalities, i.e., TG4-F and TG5. Proteins associated with the cardiomyopathy pathway, which we interpret as a sign of heart tissue damage, showed a highly specific up-regulation in those patients succumbing to the disease and were identified as death markers present in TG4-F days/weeks before death (Fig. 5A, highlighted in orange). Protein trajectories (for example MYH7, TMP1, and ACTB) belonging to these two pathways can be found in fig. S6. Of note: MYH7 is a heart tissue–specific myosin heavy chain.

Collateral damage markers due to prolonged inflammation were also observed, with an increase in TG4-S, TG4-F, and TG5 at the last time point in the most severe cases (Fig. 5A, highlighted in brown). These markers include the ECM organization and degradation pathways (MMP14, MMP2, and ELN; figs. S5 and S6), protein digestion and absorptions, and advanced glycation end products (AGE)–Receptor for AGEs (RAGE) signaling pathways. Last, cell adhesion molecules and cholesterol metabolism pathways were observed to increase in all TGs (Fig. 5A, highlighted in purple), apart from TG5, suggesting potential recovery markers.

The longitudinal sampling of the IMPACC study and its large enrollment numbers provided us with the unique opportunity to take a closer look at the survival versus death in a subcohort with similar symptomatology during the first 28 days of hospitalization. For example, we were able to study the sequence of pathophysiological events leading to death. To this end, we conducted *t* tests comparing TG4-S to TG4-F at admission, week 1, week 2, week 3, and week 4 (fig. S7). At admission, no significant differences were observed. However, individuals in TG4-F showed abundance increases of indicators of poor outcomes, including H1.5 and Osteopontin (SPP1), as early as during the first week after admission (Fig. 5C). Within 2 weeks of admission, we can observe an increase in cardiac tissue damage markers such as MYH7. The next process set in motion is ECM degradation as exemplified by the up-regulation of MMP2 between weeks 2 and 3 after admission. Toward the end of the 4-week sampling period, markers sustained acute inflammation (SAA1) increase.

### Prognostic plasma protein biomarkers for severity escalation and outcome

**Fig. 6.**
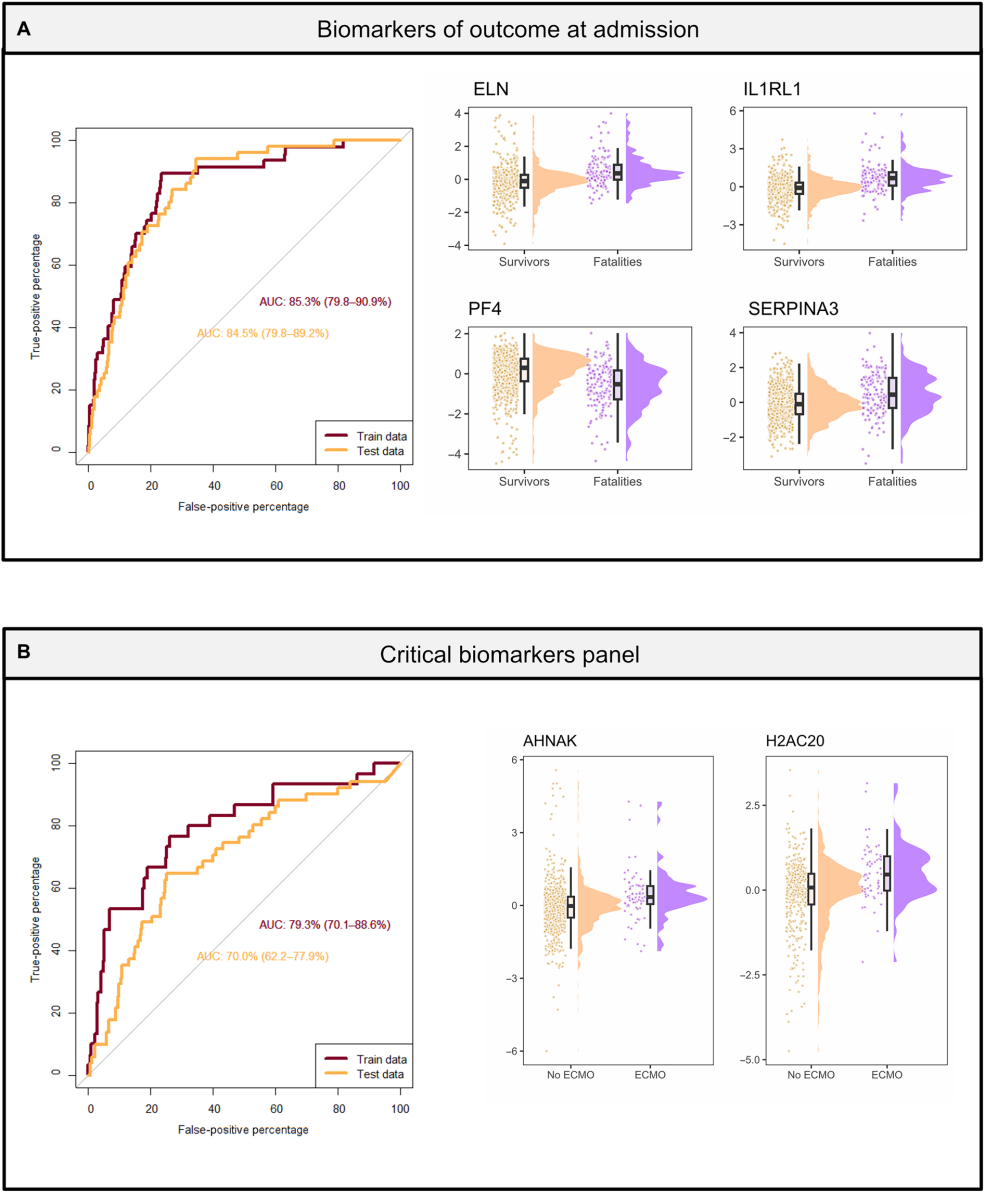
Prognostic biomarkers upon admission for outcome including use of ECMO or invasive mechanical ventilation. Predictive biomarkers at admission of outcome and escalation of care (receipt of ECMO or invasive mechanical ventilation). For both analysis, data were split into a train and test dataset. Both datasets are independent as samples were collected in different hospitals. The samples from the training dataset were collected at the following hospitals: UA-Tucson, Baylor, Boston/BWH, Case Western, OUHSC, UCLA, and Yale. The samples from the test dataset were collected at the following hospitals: Drexel/Tower Health, Emory, UF, ISMMS, OHSU, Stanford, UCSF, and UT Austin. (**A**) Panel of biomarkers for outcome with an AUROC value of 0.0.85 for the test dataset. Expression levels of the biomarker panel are composed of ELN, IL1RL1, PF4, and SERPINA3. (**B**) Panel of biomarkers for ECMO with an AUROC value of 0.77 for the test dataset. Expression levels of the biomarker panel are composed of AHNAK and H2AC20.

The large number of enrolled patients of similar symptomatology allowed us to mine the deep plasma proteomics data across thousands of proteins for biomarker panels within the “intended use population”, i.e., patients with similar symptomatology but different later outcomes, to prognosticate the clinical disease course and outcomes for hospitalized patients with SARS-CoV-2; this analysis highlights the potential to translate our proteomics findings into the clinic. Early prognostication of disease outcome or escalation of care among critically ill patients with higher mortality risk, is clinically relevant especially in resource-limited situations such as pandemics and/or rural settings. To model these two clinically relevant questions, we used a logistic regression at time of admission. Our large dataset with plasma proteome maps from 1117 individuals originating from 15 clinical sites was split into training and test datasets for validation. When splitting the samples into the two sample sets, we ensured that each sample set was composed of samples from two independent groups of hospitals, i.e., training and test sets are truly independent even based on Food and Drug Administration (FDA) standards. The dataset identified a panel of four proteins as prognostic biomarkers for disease outcome at admission. This panel, which included ELN, Interleukin 1 Receptor Like 1 (ILRL1), PF4, and SERPINA3, predicted death at time of hospital admission with an area under the receiver operating characteristic curve (AUROC) value of 0.85 for the training set and 0.85 for the test set (Fig. 6A and table S7A).

Similarly, two proteins were identified as biomarker panel at admission to prognosticate escalation of care, which we define as need for extracorporeal membrane oxygenation (ECMO) and/or invasive mechanical ventilation during the hospitalization. These proteins included AHNAK and H2AC20. Using the same training/test strategy as described above, this panel was predictive of escalation of care with an AUROC value of 0.79 for the training set and 0.70 for the test set (Fig. 6B and table S7B). By understanding immuno-protein parameters associated with distinct disease outcomes, we hope to highlight opportunities for improved therapeutic approaches in hospitalized patients.

## DISCUSSION

In this study, we examined changes in the plasma proteome of 1117 hospitalized patients with COVID-19 during the acute phase of the disease. To capture a comprehensive view, we collected up to six plasma samples from each patient within the first 28 days of hospitalization. This study design resulted in a remarkable dataset of 2934 longitudinal plasma samples, making it one of the largest cohorts of plasma samples from hospitalized patients with COVID-19, that were analyzed by LC-MS–based high-throughput deep plasma proteomics (Fig. 1A). To effectively analyze thousands of plasma samples, we advanced our published perchloric acid–based plasma protein depletion method (*42*) to be high throughput compatible and to robustly enable the detection of the lesser abundant tissue leakage proteins in plasma (*45*) in thousands of plasma samples (*44*).

The coupling of an Evosep One LC with a timsTOF PRO 2 MS has proven robust over the course of the study comprising 2934 samples (including reference samples) analyzed in three batches over a 10-month period (Fig. 1A). The detection and quantification of 2910 proteins in plasma enabled for the characterization of the host response to SARS-CoV-2 infection at admission and in the context of five clinical TGs based on disease severity (Fig. 3). In this analysis, we observed notable differences between hospitalized patients with short hospital stay versus those with delayed recovery with or fatal outcome. We detected SARS-CoV-2 NP (Fig. 2) validating the utility of our perchloric acid depletion protocol for high-throughput deep discovery plasma proteomics.

While the abundance levels of NP only showed minor severity-dependent differences, the observation frequencies showed clear time- and severity-dependent changes. At admission, NP was detected at a higher frequency (30 to 40%) in the most severe COVID-19 cases (i.e., TG4 and TG5) and was only detected in one sample (TG5 at week 4) after 2 weeks of hospitalization. NP is an early marker of severe disease in plasma from hospitalized patients with COVID-19 (*50*) as it is almost exclusively detected during the first week after hospital admission (Fig. 2C). These findings are supported by recent studies that reported that plasma viral RNA also showed similar associations with disease severity and increased risk of mortality (*51*, *52*) and detection range between 9.4 and 74.1% (*53*). The origin of plasma viremia is not known; it is hypothesized to originate from pulmonary spillage, a result of endothelial cell SARS-CoV-2 (*51*).

At admission, the plasma proteome of patients with severe COVID-19 is characterized by an increased innate immune response. Most notably, we observed an increase of the complement cascade in TG2 to TG5 (Fig. 3) consistent with other proteomics studies (*2*, *5*, *10*) (comprehensive review of current COVID plasma proteome literature is found in table S1), which have reported similar increased complement activation in COVID-19-associated but not in non–COVID-19 respiratory failure (*54*). The complement system, evolutionary conserved and nonredundant component of innate immune system, functions to detect and remove invading pathogens in the blood and interstitial fluid (*55*). Although the complement system is protective in SARS-CoV-2 infection, its hyperactivation is part of the pathophysiology of COVID-19 (*30*), illustrating that limited complement activation is beneficial in response to the virus, while hyperactivation of the complement system propagates the disease and is associated with increased disease severity.

The IMPACC study design allowed for the investigation of both the cross-sectional baseline data at hospital admission and the time course of pathways stratified by disease severity during the hospitalization (Fig. 4). The significance of longitudinally collected samples becomes evident in our analysis, as initial values often exhibit only slight variations. However, the observed temporal changes swiftly enable clear differentiation between the TGs.

Our data show that 10 days after admission, the sustained up-regulation of the complement system distinguishes TG5 from other severe COVID-19 cases (Fig. 4). Delayed and sustained complement up-regulation is specific to fatal outcome (TG5, <28 days) and thus might be indicative of imminent death (Fig. 5 and figs. S5 and S6). As sustained activation of the complement system leads to endothelial injury and prothrombic injury (*54*), timely intervention is essential. To the extent that complement activation contributes to detrimental COVID-19 outcomes, our data suggest that therapies targeting the complement system (*56*) should be administered within 10 days of hospital admission to be efficacious.

To gain a deeper understanding of disease heterogeneity associated with fatal outcome, we split TG4 into a survivor and a fatal group (TG4-S and TG4-F). We considered TG4-F distinct from TG5, i.e., we differentiate fatal outcome within 28 days to fatal outcome after 28 days. We performed an analysis comparing the first and last samples collected per individual, where we observed markers of survival (TG4-F and TG5) and markers of collateral damage/prolonged inflammation (Fig. 5 and fig. S5). To further this analysis, we compared the sequence of events before death in TG4-F in comparison with TG4-S. We noticed histone H1.5 is, with SPP1, the first protein to increase in fatal COVID-19 (TG4-F, death after >28 days). H1.5 increases during the first week after hospital admission (Fig. 5B).

Consistent with our analyses of H1.5 expression in relation to death, we observed a steady up-regulation of circulating histone (H1.5) in fatal-outcome patients (TG5) while in survivors the expression of H1.5 decreases over time (fig. S6). Ten days after hospital admission, the levels of circulating H1.5 in TG5 are higher than that of TG4-F suggesting a potential therapeutic window targeting histones. Histones contribute to the worsening COVID-19 disease by the release of proinflammatory cytokines and vascular endothelial injury (*57*) which promotes a proinflammatory loop that can lead to sepsis. Similarly, histones are also elevated in sepsis, where circulating histones have toxic effects and can lead to multiple-organ injury and/or death (*57*, *58*). Histone H1.5 is a virus-independent early marker for fatal outcome, as we also observed its early increase in patients that succumbed to an Ebola virus infection (*42*). In that study, H1.5 levels showed better performance at prognosticating fatal outcome than the widely used Ct values (AUROC 1.0 versus 0.75).

Histones are tightly linked to NETs (*59*, *60*) and their activities as they display histones to trap pathogens. Neutrophils, via a programmed cell death called NETosis, release NETs. Composed of extracellular webs of chromatin, histones, oxidizing enzymes, and antimicrobial proteins, NETs contain, inactivate, and kill infecting pathogens. NETs are beneficial to physically trap and kill pathogens including viruses. However, uncontrolled up-regulation of NETs can lead to propagating inflammation (*61*), microvascular thrombosis, and organ damage (*62*), which is consistent with the observed up-regulation of markers of cardiomyopathy, i.e., viral-infection induced severe heart damage.

While H1.5 is a prototypic NET-associated protein, several NET-associated proteins are up-regulated in severe COVID-19 (TG4 and TG5) and distinguish severe from fatal outcome (TG4 versus TG5 as well as TG4-S and TG4-F) as early as at time of admission (Figs. 3 and (5, A to C). This observation suggest that NETs are up-regulated as a first sign of potentially fatal outcome and that it might be even more deleterious than the complement system. Both features make NET-associated proteins potential early prognostic markers of survival versus death. These observations and interpretations are consistent with previous studies on NETs in COVID-19, which reported that in severe COVID-19, NETs are released (*31*) in a severity-dependent manner (*63*), and contribute to lung injury and immunothrombosis (*64*). NETs released into the intravascular circulation may bind to the vessel walls and contribute to COVID-19 associate coagulopathy.

Our observations in fatal Ebola as well as COVID-19 infections raises the possibility that histones and NETs may contribute to in fatal viral infection and could potentially be a relevant target for prognostication and/or therapy. For example, the proteins S100A8/S100A9 (aka calprotectin), which are markers of NET release, and which we found to be up-regulated in fatal COVID-19, are targets for Paquinimod, which inhibits S100A8/S100A9, which is involved inflammation and immune regulation (*65*), and has been shown to be beneficial in mice infected with SARS-CoV-2 (*66*). Paquinimod, a small-molecule immunomodulator, requires further research into its COVID treatment safety and efficacy.

Patients with critical COVID-19 are characterized by a hyperinflammatory state and aberrant inflammatory response (*67*). Patients with fatal COVID-19 (TG5) appear to be trapped in a self-amplifying inflammation loop (*68*), in which each pathway promotes the next in a positive feedback loop: complement system (hyper)activation, NETosis, and coagulopathy. As a direct consequence, the patients with the most severe COVID-19 (TG4-S, TG4-F, and TG5) demonstrated a clear temporal up-regulation of the (re)organization and degradation of the ECM (Fig. 5). In contrast, TG1 to TG3 show a decrease of these pathways over time. Upon inflammation and injury, the level of MMPs increases (*69*, *70*), illustrated by the MMP14 (see fig. S6). MMPs mediate the degradation of ECM components (*71*). Degradation of the ECM in combination with decreasing cell adhesion molecules (Fig. 4) leads to tissue destruction and fibrosis (*72*). Notably, COVID-19 pulmonary fibrosis is a complication of the disease (*72*). Hyperinflammatory state and the combined effect of complement activation, dysregulated NETosis, and endothelial injury (*73*) may contribute to major organ damage (*64*) and/or death. Notably, we found that the cardiomyopathy pathway increased over time specifically in fatal outcome i.e., TG4-F and TG5 (Figs. 4 and (5); this increase is not seen in the TG4-S group, which show a similar acute clinical trajectory as the TG4-F group, but with a nonfatal outcome. We found an up-regulation of the MYH7 proteins in fatal outcome (Fig. 5C) as early as 2 weeks in TG4-F. MYH7 is a heart-specific protein that contributes to heart muscle contraction (*74*). Our study thus provides additional evidence to the mounting evidence of long-term heart damage caused by COVID-19 (*75*–*77*). In our previous study on Ebola virus disease, we did not observe an increase in heart marker damage in fatal cases. Another set of organ damage-associated plasma proteome changes point to the liver (Fig. 4, E and F). The recovery of the liver is clearly observable in the longitudinal trajectories of cholesterol metabolism associated proteins: a steady increase for the mild COVID-19 cases (TG1 to TG3) contrasted by a delayed increase or even an initial further decrease for the severe COVID-19 cases (TG4 and TG5, respectively).

Several studies have described age, sex, and BMI as conditions for increased severity of COVID-19 disease (*78*–*80*) (table S4). Other risk factors for COVID-19 mortality that are associated with aging and inflammaging, include obesity, diabetes, and high blood pressure (*81*, *82*). Our cohort showed that increased COVID-19 severity in TG5 coincided with increased age (mean = 68.4 years versus 58.3 years in the entire cohort), and a sex bias toward men (male = 66.3% versus 59.9% in the entire cohort; see table S4 for more details). In our proteomic analysis, we observed an increase in AGE-RAGE signaling pathways in the most severe TGs (TG4-S, TG4-F, and TG5) as seen in Fig. 5. While AGE is involved in the pathogenesis of age-related diseases contributing to micro- and macrovascular complications (*83*), the RAGE is primarily expressed by epithelial cells in the alveolar sac important for SARS-CoV-2–associated hyperinflammation and lung injury (*84*, *85*). AGE-RAGE signaling molecules may contribute to COVID-19 exacerbation (*85*), due to the impact of AGE on inflammaging.

In addition, severe SARS-CoV-2 targets endothelial cells and blood vessels (*86*–*88*). Endothelial cell damage contributes to a generalized proinflammatory state which promotes complement and neutrophil activation. Effector neutrophils activate the complement system and a hypercoagulable state with endothelial injury and microthrombosis begins. If not controlled, this is exacerbated by a hyperinflammatory response, perpetuating an inflammatory loop that leads to tissue damage (*88*). In contrast, hospitalized patients with less severe COVID-19 (TG1 to TG3) are better able to control inflammation (Fig. 4, E and F). Less severe COVID-19 is associated with increased cholesterol metabolism as represented by the increase of five different apolipoproteins (Fig. 3C). In contrast, decreased levels of apolipoproteins (Figs. 3C, 4, and 5) are associated with acute severe COVID-19 (*9*) consistent with decreased plasma apolipoprotein concentrations in fatal cases of Ebola infection (*42*). These observations are consistent with reports of association of sepsis with decreased plasma apolipoproteins concentrations (*89*). We observed that these decreased apolipoprotein levels are seen as early as at the time of hospitalization.

Last, to understand potential prognostic biomarkers of disease outcome and escalation of care among our hospitalized patient population, we used logistic regression on the admission dataset.

Other studies have focused on finding early biomarkers predicative of outcome using multi-OMICS (*90*) or proteomics (*2*–*6*, *90*). While these studies found great predictive values with AUROC >0.8, they were limited to a maximum of a hundred participants and a few clinical sites. Here we proposed a set of biomarkers generated from over a thousand samples coming from 15 clinical centers that can prognosticate fatal outcome with an AUROC = 0.85 and receipt of ECMO or other invasive mechanical ventilation, i.e., the most severe form of care escalation, with an AUROC = 0.70 to 0.79. These two panels of proteins can help clinicians direct care in resource-limited pandemic settings, i.e., triaging patients, assigning hospice care, and/or identifying those patients in the need for ECMO to transfer them to a hospital with available ECMO equipment (Fig. 6). The proteins constituting our biomarker panels are linked to acute phase, inflammation, NETs, and heart failure. SERPINA3 acute phase protein is involved in inflammation. SERPINA3 (*2*, *4*–*6*, *90*) was found in other studies as indicators of COVID-19 severity. H2AC20 is a core histone and could be a marker of NETs (*91*). IL1RL1 is linked to heart failure and could be a marker of early heart damage in severe COVID-19 (*92*). While these biomarkers enable the differentiation of outcomes and the escalation of care, their integration into routine clinical tests will require validation studies using samples from patients that succumbed to viral infections.

Although unique and impactful, our study has limitations. As it is based on a hospitalized patient population our data are biased toward those that are most severe and have different comorbidities. Also, samples were collected before vaccines were available and patient were only infected with the original strain of SARS-CoV-2. Furthermore, because this was a surveillance cohort study, no healthy controls have been enrolled. During the early phase of the COVID-19 pandemic, clinical sites only permitted essential staff at the clinics and prohibited visits for pure research purposes, which hindered the collection of healthy donors. However, this lack of healthy controls is less of an issue for the overall objective of the IMPACC study as we were tasked with mapping the severity-dependent immunophenotypes of a wide range of hospitalized patients with COVID-19. Most of these limitations are overcome by longitudinal sampling allowing each patient to act as their own control, enabling the monitoring of individual trajectories instead of relying on cross-sectional snapshots. Furthermore, we were able to map the changes in the proteome leading to death, after 28 days, in a severity-matched group providing unique insight into the molecular events that precede death. Last, because of the clinical nature of the study design, the MS mode needed to be defined at the start of the study committing us to Data Dependent Aquisition-Parallel Accumulation–SErial Fragmentation (DDA-PASEF) mode.

### Conclusion

Using our recently developed biochemical plasma depletion method compatible with high-throughput deep plasma proteomics, we mapped the proteomes of 2934 plasma samples from 1117 hospitalized patients with COVID-19 enrolled in the NIAID/NIH-funded IMPACC study. The depletion method, fully validated on these 2934 plasma samples enabled a deeper characterization of the plasma proteome, including detection of SARS-CoV-2–derived NP with a severity-dependent observation frequency.

These 1117 patients had been stratified into five clinical TGs based on time to recovery/discharge and/or outcome by the IMPACC consortium (*40*). In line with the overarching objective of the IMPACC study, i.e., providing insights into the immunophenotypic heterogeneity among hospitalized patients with COVID-19 of different severities, our analysis identified not only COVID-19 severity–dependent plasma proteome changes at admission but also their temporal changes up to 28 days during the hospitalization. We demonstrate that trajectories can be more informative than baseline values at admission, highlighting the importance of longitudinal sampling. Furthermore, we provide insights into COVID-19 severity and subsequent collateral damage associated with morbidity. In particular, severity was associated with an increase of heart damage markers. Our data strongly suggest an important role for NETs in the inflammatory response to SARS-CoV-2.

Using the weekly resolution of the samples provided by the unique IMPACC study design, we were able to characterize the molecular changes and the sequence of events leading to death; the study design allowed us to perform this analysis in a severity-matched, i.e., symptomatic control group, providing unique insights in the proteome changes preceding death. In addition, the multicenter cohort design of the study and the large number of samples analyzed enabled us to perform a powerful biomarker analysis. Biomarker candidates (e.g., IL1RL1 and ELN) are proposed for distinguishing between survival and fatal outcome, as well as prognosticating escalation of care, in particular receipt of ECMO, which is particularly relevant in pandemic situations associated with resource limitation.

## MATERIALS AND METHODS

### Ethics statement

Ethics NIAID staff conferred with the Department of Health and Human Services Office for Human Research Protections (OHRP) regarding potential applicability of the public health surveillance exception [45CFR46.102 (*39*, *93*)] to the IMPACC study protocol. OHRP concurred that the study satisfied criteria for the public health surveillance exception, and the IMPACC study team sent the study protocol, and participant information sheet for review, and assessment to institutional review boards (IRBs) at participating institutions. Twelve institutions elected to conduct the study as public health surveillance, while three sites with prior IRB-approved biobanking protocols elected to integrate and conduct IMPACC under their institutional protocols (University of Texas at Austin, IRB 2020-04-0117; University of California San Francisco, IRB 20-30497; Case Western Reserve University, IRB STUDY20200573) with informed consent requirements. Participants enrolled under the public health surveillance exclusion were provided information sheets describing the study, samples to be collected, and plans for data deidentification, and use. Those that requested not to participate after reviewing the information sheet were not enrolled. In addition, participants did not receive compensation for study participation while inpatient and subsequently were offered compensation during outpatient follow-ups (*40*).

### Cohort and study design

The cohort and study design of the IMPACC study has been previously published (*39*, *40*). In brief, hospital in-patients 18 years and older admitted to one of the 20 U.S. hospitals (affiliated with 15 academic institutions) were enrolled in the study within 72 hours of hospital admission. Symptomatic patients with a confirmed positive SARS-CoV-2 PCR test were followed longitudinally for up to 28 days of their hospital stay. Patient outcome was followed for up to 12 months after discharge. The November 2021 data freeze of the clinical data was used for the subsequent analysis.

### Cohort demographics, time points, and data collected

All study participant data, containing all relevant deidentified variables, was collected using a secure electronic data collection form (*40*). Plasma samples were collected upon admission at the hospital, and up to five additional samples were collected during the acute phase in the hospital. Contains demographic information, including sex, median age, median BMI, and median symptom onset.

### Outcome categorization

As reported (*40*), the clinical severity of illness was assessed using a seven-point ordinal scale (OS), adapted from the World Health Organization COVID-19 and NIAID disease ordinal severity scales. The seven-point OS includes: OS1 = Not hospitalized, no limitations; OS2 = Not hospitalized, activity limitations or requires home O_2_; OS3 = Hospitalized, not requiring supplemental O_2_; OS4 = Hospitalized, requiring O_2_; OS5 = Hospitalized on noninvasive ventilation or high-flow O_2_; OS6 = Hospitalized on invasive mechanical ventilation and/or ECMO; OS7 = Death. The seven-point OS for respiratory status was calculated at each hospitalization time point. Patients were then classified into five clinical TGs based on longitudinal modeling of OS over time. A subset of the full IMPACC cohort was analyzed using plasma proteomics after depletion of the most abundant classical plasma proteins. We used the definition of “classical plasma proteins” from Anderson and Anderson (*45*) and applied the biochemical depletion method as described by Viode *et al.* (*44*). The detailed description of the clinical characteristics of the full IMPACC cohort has been reported (*40*). These include TG1 (*n* = 230) characterized by a mild respiratory disease and brief hospital stay with a largely uncomplicated hospital course, TG2 (*n* = 272) generally required more respiratory support than TG1 and had a longer hospital stay but were discharged without limitations, TG3 (*n* = 260 patients) was characterized by roughly similar respiratory support requirements and similar length of hospital stay as TG2 but generally had limitations at discharge, TG4 (*n* = 199) generally received more aggressive respiratory support and generally experienced a prolonged hospital stay, and TG5 (*n* = 98) characterized by high respiratory support requirements that progressed to mortality by day 28. For some analyses, TG4 was split into two subgroups: TG4 survivors (TG4-S) and TG4 fatalities (TG4-F). TG4-F includes participants who eventually died within the study but only after the 28-day sampling period.

### Sample preparation

Fifty microliters of neat plasma samples was diluted with 450 μl of water, and 25 μl of perchloric acid (70%) was added (*44*). After vigorous agitation, the suspension is kept at −20°C for 15 min. The suspension was centrifuged for 60 min (4°C, 3200*g*), and the supernatant is kept. The supernatant was mixed with 40 μl of 1% trifluoroacetic acid and loaded onto a μSPE HLB plate (Waters, catalog no. 186001828BA), preconditioned with 300 μl of methanol and twice with 500 μl of 0.1% trifluoroacetic acid. Proteins were eluted from the μSPE HLB plate with 100 μl of 90% acetonitrile 0.1% trifluoroacetic acid. After elution, the samples were dried using a Speedvac. The samples were resuspended with 35 μl of 50 mM ammonium bicarbonate and digested with 10 μl of trypsin (Promega, catalog no. V5280, 500 ng) overnight at 37°C. Digestion was stopped by the addition of 5 μl of 10% formic acid. The samples were stored at −80°C before LC-MS analysis.

### Sample MS data acquisition

Two microliters of tryptic peptides was loaded onto Evotip and analyzed using an EVOSEP one liquid chromatography (EVOSEP) connected to a TIMSTOF Pro (Bruker). The EVOSEP one method was the 60 sample per day (21-min gradient) and the mass spectrometer was operated in DDA-PASEF mode. Four PASEF tandem MS scans were triggered per cycle. DDA-PASEF parameters were set as follows: mass/charge ratio range, 100 to 1700; mobility (1/K0) range was set to 0.70 to 1.45 V·s/cm^2^; the accumulation and ramp time were 100 ms. Target intensity per individual PASEF precursor was set to 5000. The values for mobility-dependent collision energy ramping were set to 51 eV at an inversed reduced mobility (1/K0) of 1.45 V·s/cm^2^ and 21 eV at 0.7 V·s/cm^2^. Collision energies were linearly interpolated between these two 1/K0 values.

### Sample search

The method used for protein identification and quantification is described by van Zalm *et al.* (*41*). In brief, data were copied to a high-performance computing system (www.mghpcc.org) for which we wrote a parallelization strategy to facilitate the identification and quantification of proteins in such large LC-MS dataset (*41*) using MSFragger (*94*). This parallelization strategy allowed for data analysis, including match between runs, in less than 2 weeks of computing time. The Uniprot human protein sequences without isoforms were combined with the protein sequences of the SARS-CoV-2 virus into a single FASTA file downloaded on 27 March 2021. Methionine oxidation and protein N-terminal acetylation were set as variable modifications; no fixed modifications were specified. A maximum of three modifications was allowed during the peptide spectrum matching. A 1% false discovery rate was applied using the Philosopher toolkit (*95*). IonQuant (*96*) was used for quantification, which uses MS1 spectra to determine the relative quantification between samples. At least one ion was required for protein quantification.

### Statistical analysis

Statistical analysis was performed using R studio. Protein intensities were normalized using VSN (*97*) and log2 transformed for further analysis. ClusterProfiler (*49*) was used for the KEGG pathway enrichment analysis and ReactomePA (*98*) for Reactome. The heatmap in Fig. 3 was done using ComplexHeatmap (*99*).

To identify longitudinal associations, presented in Fig. 4, we tested whether proteins kinetics during hospitalization were different across the TGs via a generalized additive model with mixed effects (gamm4 v0.2.6) while controlling for sex and age. Proteins for which the average (intercept in the gamm4 documentation) or shape (smoothing term in the gamm4 documentation) differed between the TGs at FDR < 5% were considered significant dysregulated [detailed description in (*100*)].

Results from Fig. 5 were visualized using ClusterProfiler (*49*): After a paired *t* test and BH correction, the significant proteins for each TG were submitted to the CompareCluster function of the ClusterProfiler tool.

For the discovery of the prognostic biomarker panels analysis, missing values were imputed protein-wise with half the minimum value for each protein. Data were split between a training and a test cohort. This splitting met the FDA definition of independence the two cohorts featured samples from two independent sets of hospitals. The samples from the training cohort were collected: University of Arizona (UA)–Tucson, Baylor, Brigham and Women’s Hospital (BWH) Boston, Case Western, University of Oklahoma Health Sciences Center (OUHSC), University of California, Los Angeles (UCLA), and Yale. The samples from the test cohort were collected at: Drexel/Tower Health, Emory, University of Florida (UF), Icahn School of Medicine at Mount Sinai (ISMMS), Oregon Health & Science University (OHSU), Stanford, University of California, San Francisco (UCSF), and The University of Texas (UT) Austin.

A Mann-Whitney test was performed, only on the training cohort, between the two conditions, i.e., Death versus Survival or ECMO/invasive mechanical ventilation Yes versus No. The 20 most significant proteins were further evaluated by performing a stepwise selection applying the Akaike information criteria (*101*) to find the best prognostic biomarker panel. Only significant proteins were selected (fig. S2). For ROC analysis and AUROC calculation, pROC (*102*) was used.

## Supplementary Materials

### This PDF file includes:

IMPACC Network Authorship List Figs. S1 to S7 Tables S3 and S4 Legends for tables S1, S2, and S5 to S7
