# Supplementary material for: Longitudinal plasma proteomic analysis of 1117 hospitalized patients with COVID-19 identifies features associated with severity and outcomes
Source: Sci Adv. 2024 May 24;10(21):eadl5762. doi: 10.1126/sciadv.adl5762 (PMC11122669; doi:10.1126/sciadv.adl5762)
Supplement: Supplementary file 1 — IMPACC Network Authorship List Figs. S1 to S7 Tables S3 and S4 Legends for tables S1, S2, and S5 to S7 [file sciadv.adl5762_sm.pdf]

Science Advances
AAAS

Supplementary Materials for

Longitudinal plasma proteomic analysis of 1117 hospitalized patients with COVID-19 identifies features associated with severity and outcomes

Arthur Viode *et al.*

Corresponding author: Hanno Steen, hanno.steen@childrens.harvard.edu

Sci. Adv. **10**, eadl5762 (2024)
DOI: 10.1126/sciadv.adl5762

The PDF file includes:

IMPACC Network Authorship List
Figs. S1 to S7
Tables S3 and S4
Legends for tables S1, S2, and S5 to S7

Other Supplementary Material for this manuscript includes the following:

Tables S1, S2, and S5 to S7

IMPACC Network Authorship List

Authors:

Arthur Viode[1,2*], Kinga K. Smolen[2,4*], Patrick van Zalm[1,2,3*], David Stevenson[1], Meenakshi Jha[1], Kenneth Parker[1], IMPACC Network, Ofer Levy[2,4,5], Judith A. Steen[2,6,7], Hanno Steen[1,2,4,7]

* Authors contributed equally

Affiliations:

1 Department of Pathology, Boston Children’s Hospital, Boston, MA, USA

2 Harvard Medical School, Boston, MA, USA

3 Department of Neuropsychology and Psychopharmacology, EURON, Faculty of Psychology and Neuroscience, Maastricht University, Maastricht, The Netherlands

4 *Precision Vaccines Program*, Boston Children’s Hospital, Boston, MA, USA

5 Broad Institute of MIT & Harvard, Cambridge, MA, USA

6 F. M. Kirby Neurobiology Center, Boston Children's Hospital, Boston, MA, USA

7 Neurobiology Program, Boston Children's Hospital, Boston, MA, USA

Collaborators:

The IMPACC Network

National Institute of Allergy and Infectious Diseases, National Institute of Health, Bethesda, MD 20814, USA: Patrice M. Becker, Alison D. Augustine, Steven M. Holland, Lindsey B. Rosen, Serena Lee, Tatyana Vaysman

Clinical and Data Coordinating Center (CDCC) Precision Vaccines Program, Boston Children's Hospital, Boston, MA 02115, USA: Al Ozonoff, Joann Diray-Arce, Jing Chen, Alvin Kho, Carly E. Milliren, Annmarie Hoch, Ana C. Chang, Kerry McEnaney, Brenda Barton, Claudia Lentucci, Maimouna D. Murphy, Mehmet Saluvan, Tanzia Shaheen, Shanshan Liu, Caitlin Syphurs, Marisa Albert, Arash Nemati Hayati, Robert Bryant, James Abraham, Sanya Thomas, Mitchell Cooney

Benaroya Research Institute, University of Washington, Seattle, WA 98101, USA: Matthew C. Altman, Naresh Doni Jayavelu, Scott Presnell, Bernard Kohr, Tomasz Jancsyk, Azlann Arnett

La Jolla Institute for Immunology, La Jolla, CA 92037, USA: Bjoern Peters, James A. Overton, Randi Vita, Kerstin Westendorf

Knocean Inc. Toronto, ON M6P 2T3, Canada: James A. Overton

Precision Vaccines Program, Boston Children's Hospital, Harvard Medical School, Boston, MA 02115, USA: Ofer Levy, Hanno Steen, Patrick van Zalm, Benoit Fatou, Kinga K. Smolen, Arthur Viode, Simon van Haren, Meenakshi Jha, David Stevenson

Brigham and Women's Hospital, Harvard Medical School, Boston, MA 02115, USA: Lindsey R. Baden, Kevin Mendez, Jessica Lasky-Su, Alexandra Tong, Rebecca Rooks, Michael Desjardins, Amy C. Sherman, Stephen R. Walsh, Xhoi Mitre, Jessica Cauley, Xiofang Li, Bethany Evans, Christina Montesano, Jose Humberto Licona, Jonathan Krauss, Nicholas C. Issa, Jun Bai Park Chang, Natalie Izaguirre

Metabolon Inc, Morrisville, NC 27560, USA: Scott R. Hutton, Greg Michelotti, Kari Wong

Prevention of Organ Failure (PROOF) Centre of Excellence, University of British Columbia, Vancouver, BC V6T 1Z3, Canada: Scott J. Tebbutt, Casey P. Shannon

Case Western Reserve University and University Hospitals of Cleveland, Cleveland, OH 44106, USA: Rafick-Pierre Sekaly, Slim Fourati, Grace A. McComsey, Paul Harris, Scott Sieg, Susan Pereira Ribeiro

Drexel University, Tower Health Hospital, Philadelphia, PA 19104, USA: Charles B. Cairns, Elias K. Haddad, Michele A. Kutzler, Mariana Bernui, Gina Cusimano, Jennifer Connors, Kyra Woloszczuk, David Joyner, Carolyn Edwards, Edward Lee, Edward Lin, Nataliya Melnyk, Debra L. Powell, James N. Kim, I. Michael Goonewardene, Brent Simmons, Cecilia M. Smith,

Mark Martens, Brett Croen, Nicholas C. Semenza, Mathew R. Bell, Sara Furukawa, Renee McLin, George P. Tegos, Brandon Rogowski, Nathan Mege, Kristen Ulring, Pam Schearer, Judie Sheidy, Crystal Nagle

MyOwnMed Inc., Bethesda, MD 20817, USA: Vicki Seyfert-Margolis

Emory School of Medicine, Atlanta, GA 30322, USA: Nadine Rouphael, Steven E. Bosinger, Arun K. Boddapati, Greg K. Tharp, Kathryn L. Pellegrini, Brandi Johnson, Bernadine Panganiban, Christopher Huerta, Evan J. Anderson, Hady Samaha, Jonathan E. Sevransky, Laurel Bristow, Elizabeth Beagle, David Cowan, Sydney Hamilton, Thomas Hodder, Amer Bechnak, Andrew Cheng, Aneesh Mehta, Caroline R. Ciric, Christine Spainhour, Erin Carter, Erin M. Scherer, Jacob Usher, Kieffer Hellmeister, Laila Hussaini, Lauren Hewitt, Nina Mcnair, Susan Pereira Ribeiro

Icahn School of Medicine at Mount Sinai, New York, NY 10029, USA: Ana Fernandez-Sesma, Viviana Simon, Florian Krammer, Harm Van Bakel, Seunghee Kim-Schulze, Ana Silvia Gonzalez-Reiche, Jingjing Qi, Brian Lee, Juan Manuel Carreño, Gagandeep Singh, Ariel Raskin, Johnstone Tcheou, Zain Khalil, Adriana van de Guchte, Keith Farrugia, Zenab Khan, Geoffrey Kelly, Komal Srivastava, Lily Q. Eaker, Maria C. Bermúdez-González, Lubbertus C.F. Mulder, Katherine F. Beach, Miti Saksena, Deena Altman, Erna Kojic, Levy A. Sominsky, Arman Azad, Dominika Bielak, Hisaaki Kawabata, Temima Yellin, Miriam Fried, Leeba Sullivan, Sara Morris, Giulio Kleiner, Daniel Stadlbauer, Jayeeta Dutta, Hui Xie, Manishkumar Patel, Kai Nie

Immunai Inc. New York, NY 10016, USA: Adeeb Rahman

Oregon Health Sciences University, Portland, OR 97239, USA: William B. Messer, Catherine L. Hough, Sarah A.R. Siegel, Peter E. Sullivan, Zhengchun Lu, Amanda E. Brunton, Matthew Strnad, Zoe L. Lyski, Felicity J. Coulter, Courtney Micheleti

Stanford University School of Medicine, Palo Alto, CA 94305, USA: Holden Maecker, Bali Pulendran, Kari C. Nadeau, Yael Rosenberg-Hasson, Michael Leipold, Natalia Sigal, Angela Rogers, Andrea Fernandes, Monali Manohar, Evan Do, Iris Chang, Alexandra S. Lee, Catherine Blish, Henna Naz Din, Jonasel Roque, Linda Geng, Maja Artandi, Mark M. Davis, Neera Ahuja, Samuel S. Yang, Sharon Chinthrajah, Thomas Hagan

David Geffen School of Medicine at the University of California Los Angeles, Los Angeles CA 90095, USA: Elaine F. Reed, Joanna Schaenman, Ramin Salehi-Rad, Adreanne M. Rivera, Harry C. Pickering, Subha Sen, David Elashoff, Dawn C. Ward, Jenny Brook, Estefania Ramires- Sanchez, Megan Llamas, Claudia Perdomo, Clara E. Magyar, Jennifer Fulcher

University of California San Francisco, San Francisco, CA 94115, USA: David J. Erle, Carolyn S. Calfee, Carolyn M. Hendrickson, Kirsten N. Kangelaris, Viet Nguyen, Deanna Lee, Suzanna Chak, Rajani Ghale, Ana Gonzalez, Alejandra Jauregui, Carolyn Leroux, Luz Torres Altamirano, Ahmad Sadeed Rashid, Andrew Willmore, Prescott G. Woodruff, Matthew F. Krummel, Sidney Carrillo, Alyssa Ward, Charles R. Langelier, Ravi Patel, Michael Wilson, Ravi Dandekar, Bonny Alvarenga, Jayant Rajan, Walter Eckalbar, Andrew W. Schroeder, Gabriela K.

Fragiadakis, Alexandra Tsitsiklis, Eran Mick, Yanedth Sanchez Guerrero, Christina Love, Lenka Maliskova, Michael Adkisson, Aleksandra Leligdowicz, Alexander Beagle, Arjun Rao, Austin Sigman, Bushra Samad, Cindy Curiel, Cole Shaw, Gayelan Tietje-Ulrich, Jeff Milush, Jonathan Singer, Joshua J. Vasquez, Kevin Tang, Legna Betancourt, Lekshmi Santhosh, Logan Pierce, Maria Tecero Paz, Michael Matthay, Neeta Thakur, Nicklaus Rodriguez, Nicole Sutter, Norman Jones, Pratik Sinha, Priya Prasad, Raphael Lota, Sadeed Rashid, Saurabh Asthana, Sharvari Bhide, Tasha Lea, Yumiko Abe-Jones

Yale School of Medicine, New Haven, CT 06510, USA: David A. Hafler, Ruth R. Montgomery, Albert C. Shaw, Steven H. Kleinstein, Jeremy P. Gygi, Shrikant Pawar, Anna Konstorum, Ernie Chen, Chris Cotsapas, Xiaomei Wang, Leqi Xu, Charles Dela Cruz, Akiko Iwasaki, Subhasis Mohanty, Allison Nelson, Yujiao Zhao, Shelli Farhadian, Hiromitsu Asashima, Omkar Chaudhary, Andreas Coppi, John Fournier, M. Catherine Muenker, Allison Nelson, Khadir Raddassi, Michael Rainone, William Ruff, Syim Salahuddin, Wade L. Shulz, Pavithra Vijayakumar, Haowei Wang, Esio Wunder Jr., H. Patrick Young, Albert I. Ko, Xiomei Wang

Yale School of Public Health, New Haven, CT 06510, USA: Denise Esserman, Leying Guan, Anderson Brito, Jessica Rothman, Nathan D. Grubaugh

Baylor College of Medicine and the Center for Translational Research on Inflammatory Diseases, Houston, TX 77030, USA: David B. Corry, Farrah Kheradmand, Li-Zhen Song, Ebony Nelson

Oklahoma University Health Sciences Center, Oklahoma City, OK 73104, USA: Jordan P. Metcalf, Nelson I. Agudelo Higuita, Lauren A. Sinko, J. Leland Booth, Douglas A. Drevets, Brent R. Brown

University of Arizona, Tucson AZ 85721, USA: Monica Kraft, Chris Bime, Jarrod Mosier, Heidi Erickson, Ron Schunk, Hiroki Kimura, Michelle Conway, Dave Francisco, Allyson Molzahn, Connie Cathleen Wilson, Ron Schunk, Trina Hughes, Bianca Sierra

University of Florida, Gainesville, FL 32611, USA: Mark A. Atkinson, Scott C. Brakenridge, Ricardo F. Ungaro, Brittany Roth Manning, Lyle Moldawer

University of Florida, Jacksonville, FL 32218, USA: Jordan Oberhaus, Faheem W. Guirgis

University of South Florida, Tampa FL 33620, USA: Brittney Borresen, Matthew L. Anderson

University of Texas, Austin, TX 78712, USA: Lauren I. R. Ehrlich, Esther Melamed, Cole Maguire, Dennis Wylie, Justin F. Rousseau, Kerin C. Hurley, Janelle N. Geltman, Nadia Siles, Jacob E. Rogers.

IMPACC Network Competing Interests

The Icahn School of Medicine at Mount Sinai has filed patent applications relating to SARSCoV-2 serological assays and NDV-based SARS-CoV-2 vaccines which list Florian Krammer as co-inventor. Mount Sinai has spun out a company, Kantaro, to market serological tests for SARS-CoV-2. Florian Krammer has consulted for Merck and Pfizer (before 2020), and is currently consulting for Pfizer, Seqirus, 3rd Rock Ventures, Merck and Avimex. The Krammer laboratory is also collaborating with Pfizer on animal models of SARS-CoV-2. Viviana Simon is a co-inventor on a patent filed relating to SARS-CoV-2 serological assays (the "Serology Assays"). Ofer Levy is a named inventor on patents held by Boston Children's Hospital relatin to vaccine adjuvants and human in vitro platforms that model vaccine action. His laboratory has received research support from GlaxoSmithKline (GSK). Charles Cairns serves as a consultant to bioMerieux and is funded for a grant from Bill & Melinda Gates Foundation. James A Overton is a consultant at Knocean Inc. Jessica Lasky-Su serves as a scientific advisor of Precion Inc. Scott R. Hutton, Greg Michelloti and Kari Wong are employees of Metabolon Inc. Vicki Seyfer-Margolis is a current employee of MyOwnMed. Nadine Rouphael reports contracts with Lilly and Sanofi for COVID-19 clinical trials and serves as a consultant for ICON EMMES for consulting on safety for COVID19 clinical trials. Adeeb Rahman is a current employee of Immunai Inc. Steven Kleinstein is a consultant related to ImmPort data repository for Peraton. Nathan Grabaugh is a consultant for Tempus Labs and the National Basketball Association. Akiko Iwasaki is a consultant for 4BIO, Blue Willow Biologics, Revelar Biotherapeutics, RIGImmune, Xanadu Bio, Paratus Sciences. Monika Kraft receives research funds paid to her institution from NIH, ALA; Sanofi, Astra-Zeneca for work in asthma, serves as a consultant for Astra-Zeneca, Sanofi, Chiesi, GSK for severe asthma; is a co-founder and CMO for RaeSedo, Inc, a company created to develop peptidomimetics for treatment of inflammatory lung disease.

Esther Melamed received research funding from Babson Diagnostics, honorarium from Multiple Sclerosis Association of America and has served on advisory boards of Genentech, Horizon, Teva and Viela Bio. Carolyn Calfee receives research funding from NIH, FDA, DOD, Roche-Genentech and Quantum Leap Healthcare Collaborative as well as consulting services for Janssen, Vasomune, Gen1e Life Sciences, NGMBio, and Cellenkos. Wade Schulz was an investigator for a research agreement, through Yale University, from the Shenzhen Center for Health Information for work to advance intelligent disease prevention and health promotion; collaborates with the National Center for Cardiovascular Diseases in Beijing; is a technical consultant to Hugo Health, a personal health information platform; cofounder of Refactor Health, an AI-augmented data management platform for health care; and has received grants from Merck and Regeneron Pharmaceutical for research related to COVID-19.

A.

Time points collected	Number of participants	Cumulative number of participants	Number of samples	Cumulative number of samples
1	304	304	304	304
2	275	579	550	854
3	268	847	804	1658
4	141	988	564	2222
5	73	1061	365	2587
6+*	56	**1117**	347	**2934**

B.

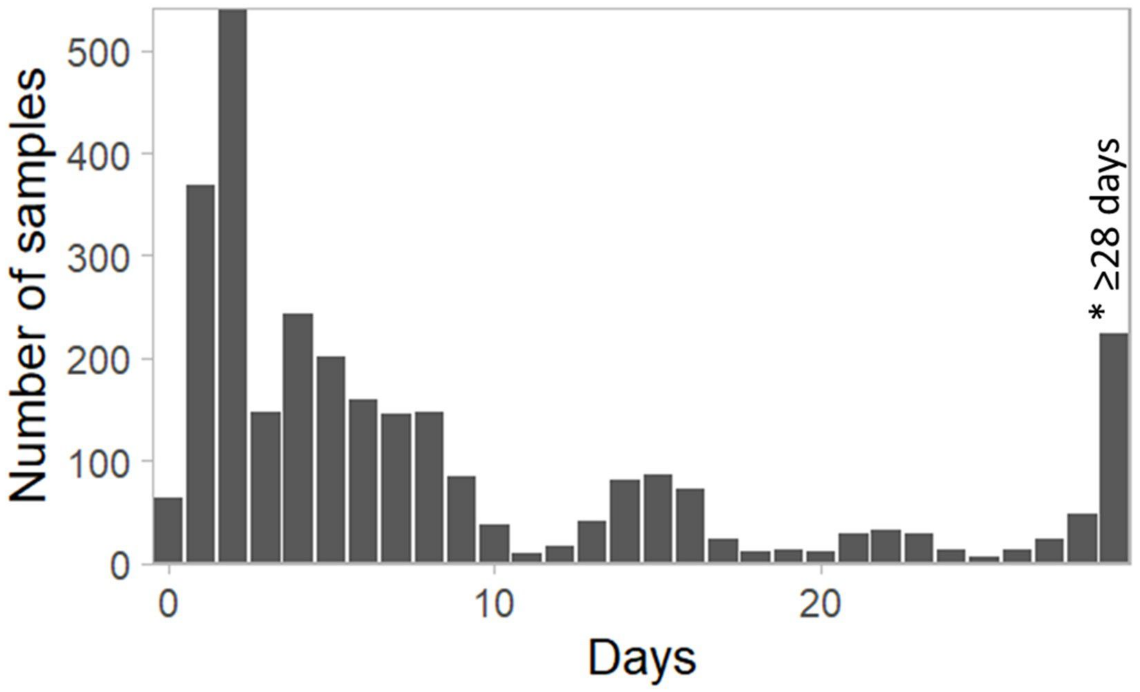

Supplementary Figure 1 A. Distribution table depicting the number of samples per visit (time point), and B. distribution graph depicting how many patients provided a sample over time.

Step 1: Independent Cohorts training and validation

Cohort A Training

Trajectory group	TG1-Full revovery (N=86)	TG2-Full recovery-Slow (N=108)	TG3-Limited recovery (N=111)	TG4-Partial recovery (N=94)	TG5-Did not recover (N=47)	Overall (N=446)
**Enrollment Site**						
Arizona	3 (3.5%)	6 (5.6%)	17 (15.3%)	14 (14.9%)	12 (25.5%)	52 (11.7%)
Baylor	6 (7.0%)	13 (12.0%)	12 (10.8%)	3 (3.2%)	4 (8.5%)	38 (8.5%)
Boston/BWH	19 (22.1%)	18 (16.7%)	15 (13.5%)	4 (4.3%)	4 (8.5%)	60 (13.5%)
Case Western	18 (20.9%)	16 (14.8%)	18 (16.2%)	4 (4.3%)	2 (4.3%)	58 (13.0%)
OUHSC (Oklahoma)	2 (2.3%)	6 (5.6%)	6 (5.4%)	15 (16.0%)	9 (19.1%)	38 (8.5%)
UCLA	16 (18.6%)	16 (14.8%)	23 (20.7%)	42 (44.7%)	8 (17.0%)	105 (23.5%)
Yale	22 (25.6%)	33 (30.6%)	20 (18.0%)	12 (12.8%)	8 (17.0%)	95 (21.3%)

Independent Cohort B Validation

Trajectory group	TG1-Full revovery (N=144)	TG2-Full recovery-Slow (N=164)	TG3-Limited recovery (N=149)	TG4-Partial recovery (N=105)	TG5-Did not recover (N=51)	Overall (N=613)
**Enrollment Site**						
Drexel/Tower Health	29 (20.1%)	26 (15.9%)	12 (8.1%)	7 (6.7%)	13 (25.5%)	87 (14.2%)
Emory	36 (25.0%)	33 (20.1%)	23 (15.4%)	10 (9.5%)	11 (21.6%)	113 (18.4%)
Florida	27 (18.8%)	23 (14.0%)	26 (17.4%)	13 (12.4%)	6 (11.8%)	95 (15.5%)
ISMMS (Mt Sinai)	6 (4.2%)	11 (6.7%)	8 (5.4%)	8 (7.6%)	2 (3.9%)	35 (5.7%)
OHSU (Oregon)	3 (2.1%)	3 (1.8%)	7 (4.7%)	11 (10.5%)	9 (17.6%)	33 (5.4%)
Stanford	23 (16.0%)	28 (17.1%)	23 (15.4%)	12 (11.4%)	2 (3.9%)	88 (14.4%)
UCSF	18 (12.5%)	36 (22.0%)	18 (12.1%)	38 (36.2%)	5 (9.8%)	115 (18.8%)
UT Austin	2 (1.4%)	4 (2.4%)	32 (21.5%)	6 (5.7%)	3 (5.9%)	47 (7.7%)

Step 2: Mann-withney test selection of top 21 features (p-adj)

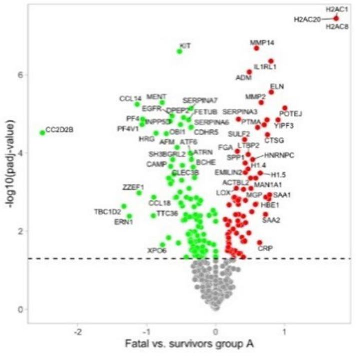

H2AC1, H2AC20,H2AC8,MMP14, KIT,IL1RL1,ADM,ELN,MENT,MMP2, CCL14,POTEJ,SERPINA7,EGFR,DPEP2,PF4, SERPINA3, ACTA1,FETUB,INPP5D,YIPF3

Step 3: GLM with step AIC to find best biomarkers

Model : glm(DX ~., data = dfA, family = 'binomial') %>% stepAIC(trace = T, direction = 'backward')

```
Call:
glm(formula = DX ~ DPEP2 + ELN + H2AC20 + H2AC8 + IL1RL1 + MENT +
    MMP2 + PF4 + SERPINA3 + SERPINA7, family = "binomial", data = dfA)

Coefficients:
             Estimate Std. Error z value Pr(>|z|)
(Intercept) -11.7916    14.0207  -0.841  0.40034
DPEP2        -0.4579     0.2542  -1.801  0.07165 .
ELN           0.4489     0.2032   2.210  0.02713 *
H2AC20      144.6098    78.1910   1.849  0.06439 .
H2AC8      -144.2445    78.1686  -1.845  0.06499 .
IL1RL1        0.4679     0.1624   2.881  0.00396 **
MENT         -0.3605     0.2026  -1.779  0.07525 .
MMP2         -0.2851     0.1879  -1.517  0.12928
PF4          -0.2875     0.1237  -2.324  0.02015 *
SERPINA3      1.3594     0.5698   2.386  0.01704 *
SERPINA7     -0.9616     0.5554  -1.731  0.08338 .
```

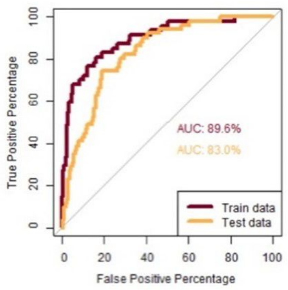

Step 4: **Final model** selection of features having a p-value <0.05

```
Call:
glm(formula = DX ~ ., family = "binomial", data = dfA)

Coefficients:
            Estimate Std. Error z value Pr(>|z|)
(Intercept) -45.3034    11.3935  -3.976    7e-05 ***
ELN           0.5698     0.1677   3.398 0.000678 ***
IL1RL1        0.5125     0.1506   3.403 0.000667 ***
PF4          -0.3551     0.1141  -3.111 0.001866 **
SERPINA3      1.5218     0.4915   3.096 0.001962 **
---
Signif. codes:  0 '***' 0.001 '**' 0.01 '*' 0.05 '.' 0.1 ' ' 1
```

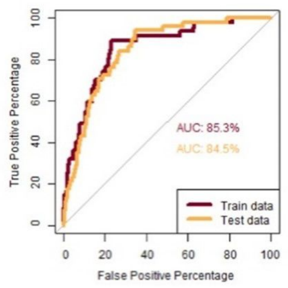

Supplementary Figure 2: Workflow for biomarker discovery. First the cohort was split into two independent sub-cohorts (based on hospitals). Then the top 20 proteins were selected for general linear model analysis with step AIC. Finally, only significant features were selected for the biomarker panel.

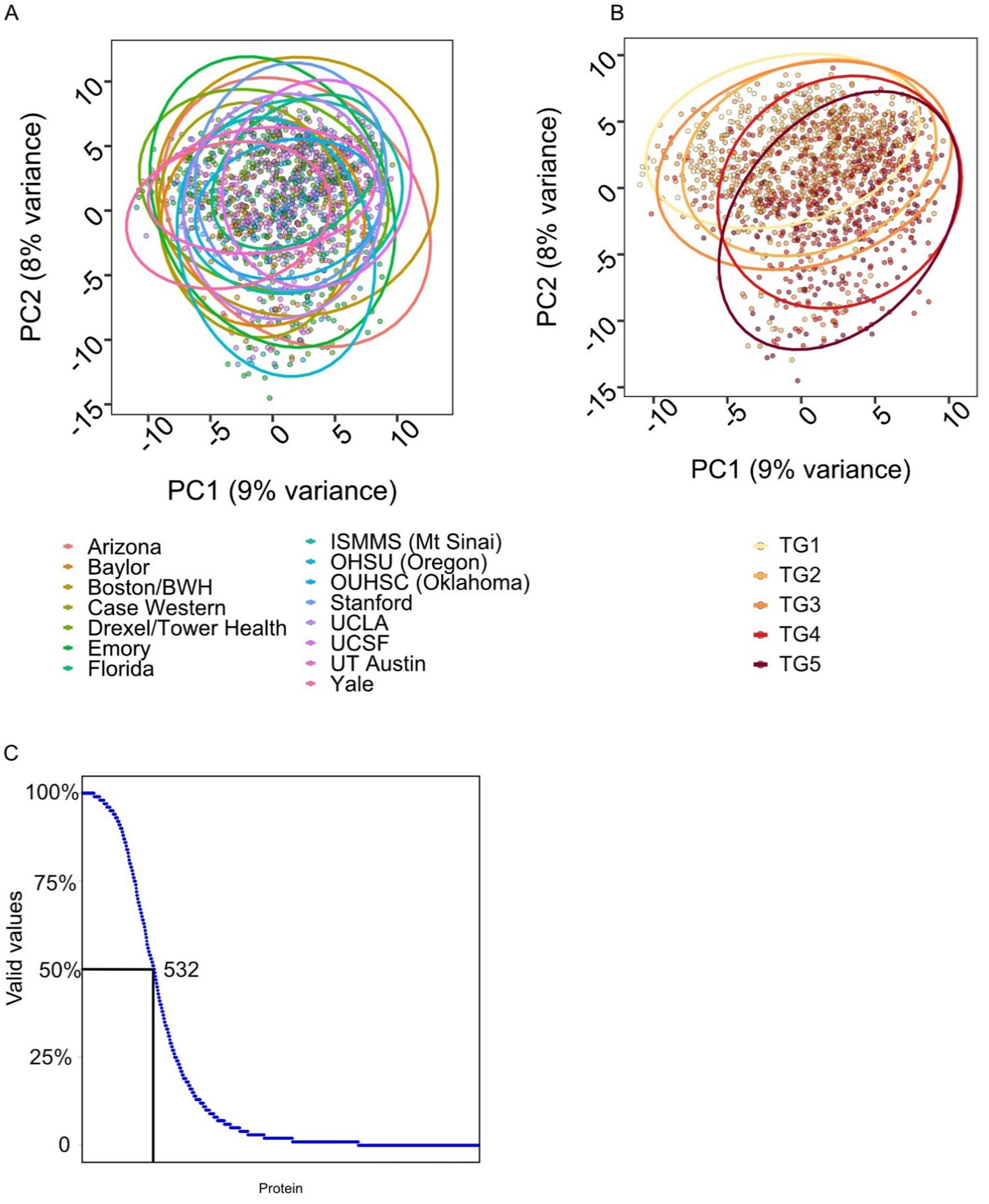

Supplementary Figure 3: A) Principal component analysis (PCA) colored-coded according to the hospital sites. B) PCA colored-coded according to the trajectory group. C) number of quantified values per protein

A.

MSDNGPQNQRNAPRITFGGPSDSTGSNQNGERSGARSKQR**RPQGL**
PNNTASWFTALTQHGKEDLKFPR**GQGVPINTNSSPDDQIGYYR**RAT
RRIRGGDGKMKDLSPRWYFYYLGTGPEAGLPYGANK**DGIIWVATEGA**
LNTPKDHIGTRNPANNAAIVLQLPQGTTLPKGFYAEGSRGGSQASSR
SSSRSRNSSRNSTPGSSRGTSPARMAGNGGDAALALLLLDRLNQLESK
MSGKGQQQQGQTVTKKSAAEASKKPRQKRTATKAYNVTQAFGRRG
PEQTQGNFGDQELIRQGTDYKHWPQIAQFAPSASAFFGMSRIGMEV
TPSGTWLTYTGAIKLDDKDPNFKDQVILLNKHIDAYKTFPPTEPKKDKK
KK**ADETQALPQR**QK**KQQTVTLLPAADLDDFSK**QLQQSMSSADSTQA

B.

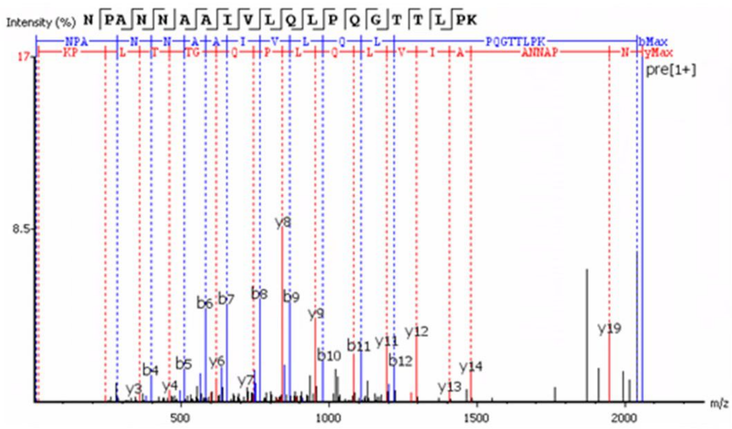

Supplementary Figure 4: A. sequence coverage of NCAP protein. B. Tandem mass spectrum of the unique NCAP-derived peptide NPANNAAIVLQLPQGTTLPK2+ (m/z= 1030.579).

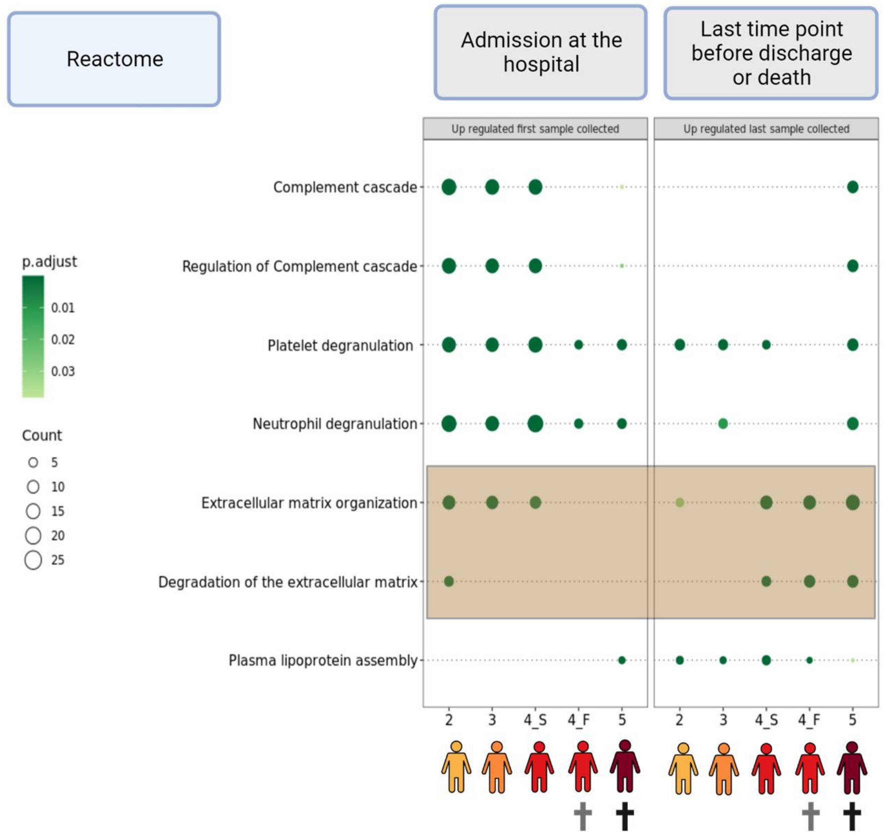

Supplementary Figure 5: Reactome Death and collateral damage

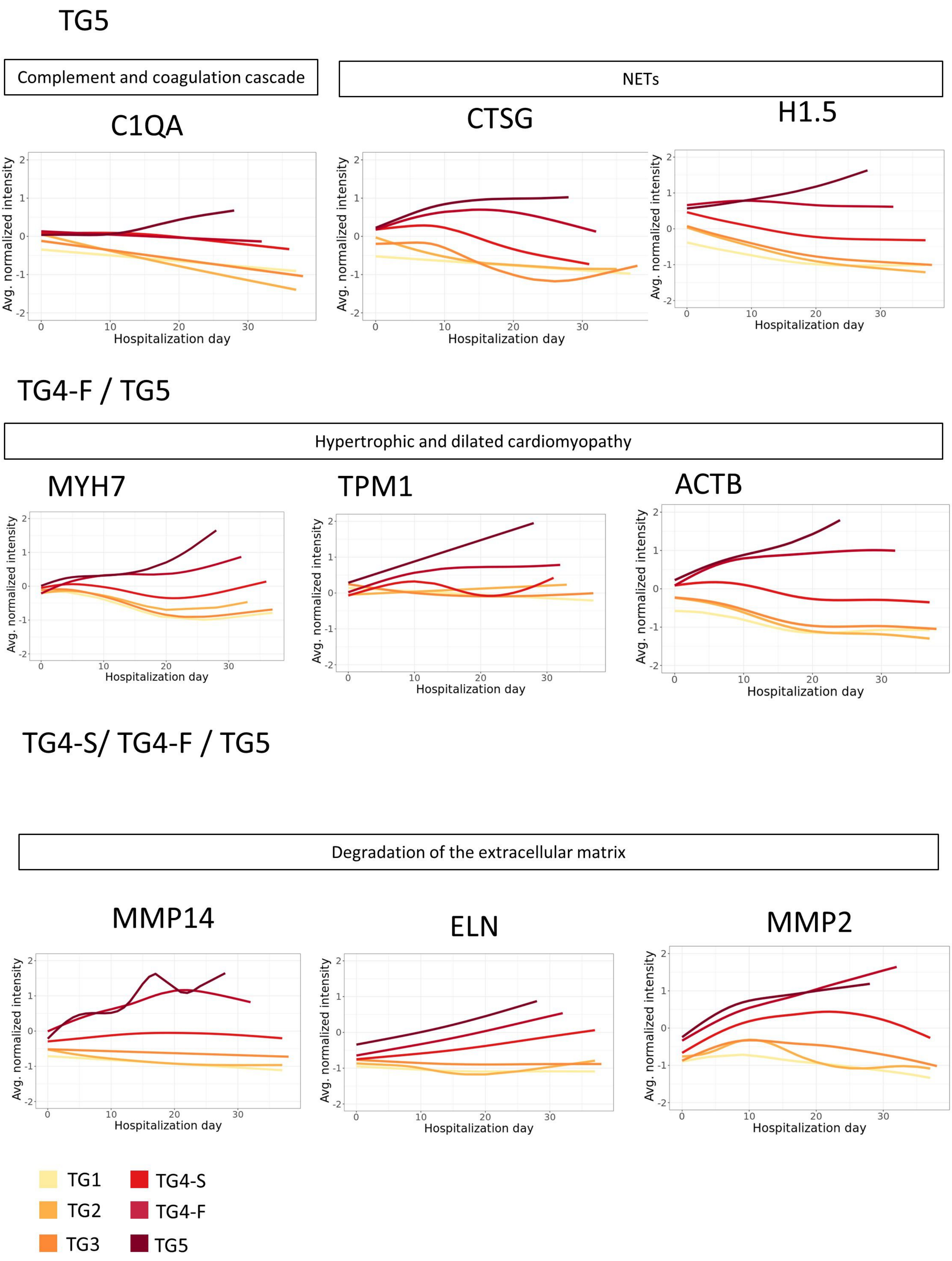

Supplementary Figure 6. Protein trajectory with TG4-F and TG4-S

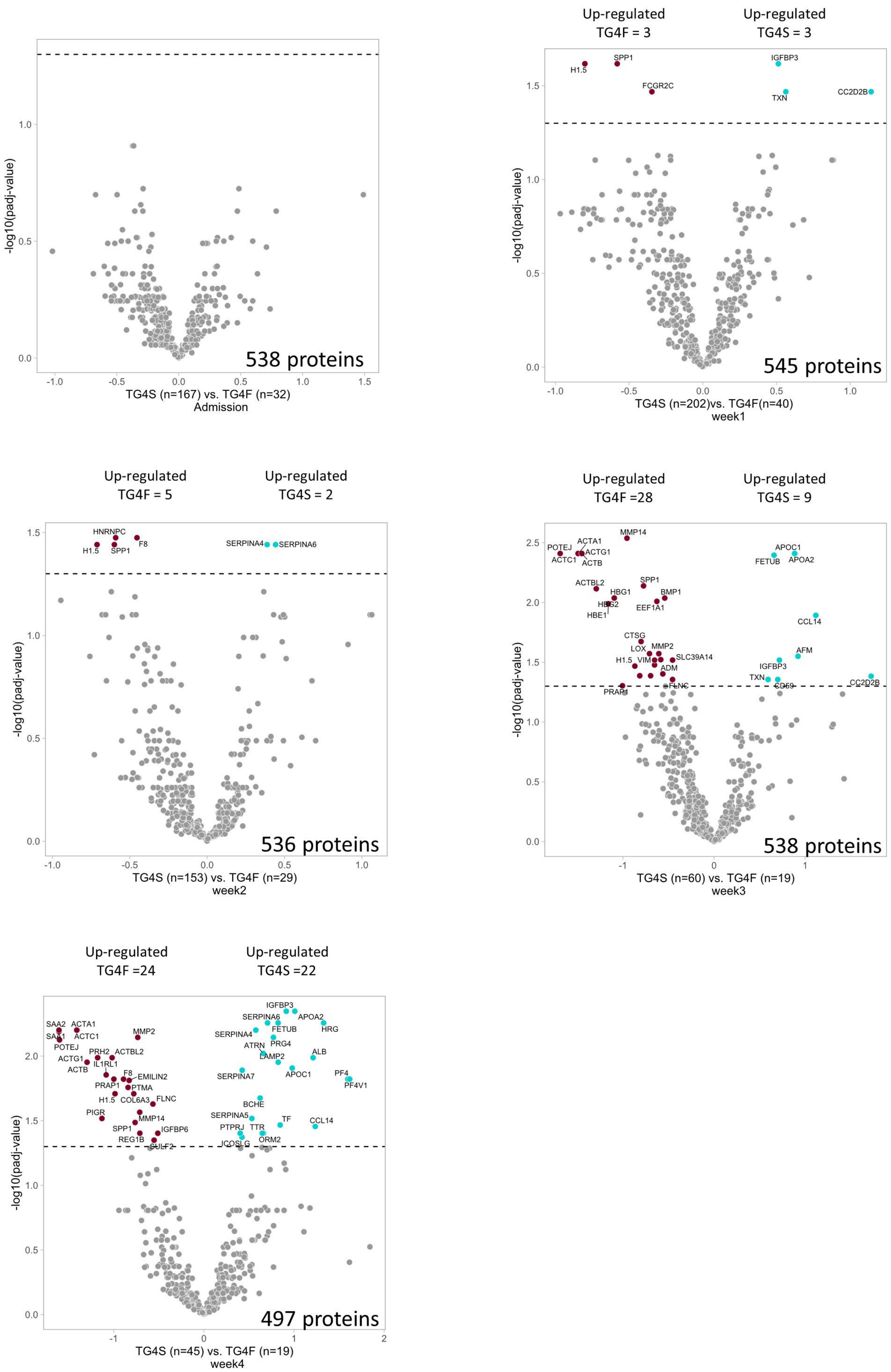

Supplementary Figure 7. T-tests comparing TG4-S to TG4-F at admission, week 1, week 2, week 3 and week 4.

Trajectory group	TG1-Full revovery (N=238)	TG2-Full recovery-Slow (N=294)	TG3-Limited recovery (N=270)	TG4-Partial recovery (N=211)	TG5-Did not recover (N=104)	Overall (N=1117)
**Enrollment Site**						
UA-Tuscson	3 (1.3%)	7 (2.4%)	19 (7.0%)	16 (7.6%)	12 (11.5%)	57 (5.1%)
Baylor	7 (2.9%)	14 (4.8%)	13 (4.8%)	3 (1.4%)	4 (3.8%)	41 (3.7%)
Boston/BWH	20 (8.4%)	19 (6.5%)	16 (5.9%)	5 (2.4%)	4 (3.8%)	64 (5.7%)
Case Western	18 (7.6%)	16 (5.4%)	18 (6.7%)	4 (1.9%)	2 (1.9%)	58 (5.2%)
Drexel/Tower Health	32 (13.4%)	28 (9.5%)	13 (4.8%)	7 (3.3%)	13 (12.5%)	93 (8.3%)
Emory	37 (15.5%)	38 (12.9%)	23 (8.5%)	11 (5.2%)	11 (10.6%)	120 (10.7%)
ISMMS (Mt Sinai)	8 (3.4%)	16 (5.4%)	11 (4.1%)	10 (4.7%)	3 (2.9%)	48 (4.3%)
OUHSC (Oklahoma)	2 (0.8%)	7 (2.4%)	6 (2.2%)	16 (7.6%)	10 (9.6%)	41 (3.7%)
Stanford	23 (9.7%)	28 (9.5%)	23 (8.5%)	13 (6.2%)	2 (1.9%)	89 (8.0%)
UCLA	16 (6.7%)	19 (6.5%)	24 (8.9%)	44 (20.9%)	11 (10.6%)	114 (10.2%)
UCSF	18 (7.6%)	38 (12.9%)	18 (6.7%)	38 (18.0%)	5 (4.8%)	117 (10.5%)
UT Austin	2 (0.8%)	5 (1.7%)	33 (12.2%)	7 (3.3%)	3 (2.9%)	50 (4.5%)
Yale	22 (9.2%)	33 (11.2%)	20 (7.4%)	13 (6.2%)	9 (8.7%)	97 (8.7%)
UF	27 (11.3%)	23 (7.8%)	26 (9.6%)	13 (6.2%)	6 (5.8%)	95 (8.5%)
OHSU	3 (1.3%)	3 (1.0%)	7 (2.6%)	11 (5.2%)	9 (8.7%)	33 (3.0%)

Supplementary Table 3: Demographic table depicting the breakdown of the participants based on their trajectory group and clinical site

Trajectory group	TG1-Full revovery (N=238)	TG2-Full recovery-Slow (N=294)	TG3-Limited recovery (N=270)	TG4-Partial recovery (N=211)	TG5-Did not recover (N=104)	Overall (N=1117)
**Sex**						
Male	135 (56.7%)	186 (63.3%)	147 (54.4%)	142 (67.3%)	70 (67.3%)	680 (60.9%)
Female	103 (43.3%)	108 (36.7%)	123 (45.6%)	69 (32.7%)	34 (32.7%)	437 (39.1%)
**Age (y)**						
Mean (SD)	54.0 (15.4)	56.1 (15.6)	58.9 (14.5)	61.1 (12.8)	68.5 (12.4)	58.4 (15.0)
Median [Min, Max]	54.5 [22.0, 87.0]	56.0 [18.0, 95.0]	59.0 [25.0, 93.0]	62.0 [18.0, 94.0]	70.0 [22.0, 96.0]	59.0 [18.0, 96.0]
**Respiratory status upon admission**						
3	137 (57.6%)	46 (15.6%)	51 (18.9%)	2 (0.9%)	9 (8.7%)	245 (21.9%)
4	87 (36.6%)	184 (62.6%)	155 (57.4%)	21 (10.0%)	18 (17.3%)	465 (41.6%)
5	9 (3.8%)	47 (16.0%)	56 (20.7%)	98 (46.4%)	36 (34.6%)	246 (22.0%)
6	1 (0.4%)	11 (3.7%)	4 (1.5%)	90 (42.7%)	41 (39.4%)	147 (13.2%)
Missing	4 (1.7%)	6 (2.0%)	4 (1.5%)	0 (0%)	0 (0%)	14 (1.3%)
**Outcome after 28 days**						
Fatalities	3 (1.3%)	6 (2.0%)	9 (3.3%)	35 (16.6%)	104 (100%)	157 (14.1%)
Survivors	235 (98.7%)	288 (98.0%)	261 (96.7%)	176 (83.4%)	0 (0%)	960 (85.9%)
**BMI**						
Mean (SD)	32.7 (8.24)	32.3 (7.53)	32.5 (9.06)	32.6 (8.16)	32.1 (8.75)	32.5 (8.29)
Median [Min, Max]	31.2 [16.5, 69.5]	31.6 [9.30, 60.0]	31.2 [15.7, 69.8]	31.4 [17.0, 64.2]	30.4 [18.5, 66.4]	31.3 [9.30, 69.8]
Missing	11 (4.6%)	15 (5.1%)	12 (4.4%)	5 (2.4%)	0 (0%)	43 (3.8%)
**Symptom onset (Days)**						
Mean (SD)	-7.38 (5.25)	-7.11 (4.76)	-9.89 (26.6)	-7.60 (5.58)	-7.78 (7.20)	-7.96 (13.6)
Median [Min, Max]	-7.00 [-31.0, 0]	-7.00 [-35.0, 0]	-7.00 [-376, 0]	-6.00 [-35.0, 0]	-6.00 [-35.0, 0]	-7.00 [-376, 0]
Missing	40 (16.8%)	40 (13.6%)	60 (22.2%)	39 (18.5%)	23 (22.1%)	202 (18.1%)

Supplementary Table 4: Demographic table depicting the characteristics of the study participants based on their trajectory groups.

Supplementary Figures and Tables:

Supplementary Figure 1: A. Distribution table depicting the number of samples per visit (time point), and B. distribution graph depicting how many patients provided a sample over time.

Supplementary Figure 2: Workflow for biomarker discovery. First, the cohort was split into two independent sub-cohorts (based on hospitals). Then the top 20 proteins were selected for general linear model analysis with step AIC. Finally, only significant features were selected for the biomarker panel.

Supplementary Figure 3: number of quantified values per protein

Supplementary Figure 4: A. sequence coverage of NCAP protein. B. Tandem mass spectrum of the unique NCAP-derived peptide NPANNAAIVLQLPQGTTLPK2+ (m/z= 1030.579).

Supplementary Figure 5: Relationship of Reactome to Death and collateral damage

Supplementary Figure 6: Protein trajectory with TG4-F and TG4-S

Supplementary Figure 7. T-tests comparing TG4-S to TG4-F at admission, week 1, week 2, week 3 and week 4.

Supplementary Table 1: Review of the current state of COVID-19 proteomics

Supplementary Table 2: Sample breakdown by week of hospitalization

Supplementary Table 3: Demographic table depicting the breakdown of the participants based on their trajectory group and clinical site.

Supplementary Table 4: Demographic table depicting the characteristics of the study participants based on their trajectory groups. Clinical Table S4 represents the individuals analysed by MS-based proteomics and is a subset of the entire IMPACC cohort.

Supplementary Table 5: Post-hoc Tukey test between TG at admission

Supplementary Table 6: p-values resulting from the smoothing spline regression analysis.

Supplementary Table 7: Biomarker panels, including the AUROC with CI
